# Infrared Thermography for Defect Detectability in Structural Health Monitoring of Concrete Structures: Heat Transfer Physics, Resolution Limits, and AI-Assisted Diagnostics

**DOI:** 10.3390/ma19122596

**Published:** 2026-06-16

**Authors:** Wiktor Wciślik, Sylwia Wciślik

**Affiliations:** 1 Department of Strength of Materials and Structures Diagnostics, Kielce University of Technology, al. Tysiąclecia Państwa Polskiego 7, 25-314 Kielce, Poland; wwcislik@tu.kielce.pl; 2 Department of Building Physics and Renewable Energy, Kielce University of Technology, al. Tysiąclecia Państwa Polskiego 7, 25-314 Kielce, Poland

**Keywords:** infrared thermography, structural health monitoring, defect detectability, reinforced concrete, thermal diffusivity, thermal contrast, active thermography, artificial intelligence

## Abstract

Infrared thermography (IRT) has emerged as a vital tool for structural health monitoring (SHM), yet the quantitative links between heat transfer physics and defect detectability in reinforced concrete remain underdeveloped. This review establishes a physics-oriented framework to clarify the mechanisms, operational parameters, and technological constraints governing IRT-based diagnostics. We analyze the interplay of thermal diffusivity, emissivity, and transient conduction in generating defect-induced contrasts. Quantitative synthesis identifies a minimum thermal contrast of 0.5 °C as a requirement for reliable detection, with active thermography proving effective for defects within a 5–10 cm depth range. Furthermore, we quantify how moisture-induced emissivity variations exceeding 10% introduce critical uncertainties in field measurements. The study underscores the necessity of integrating heat transfer theory with advanced signal processing and artificial intelligence (AI) to overcome current resolution limits. By defining these physical boundaries, this review provides a robust foundation for automated, intelligent infrastructure diagnostics, emphasizing that detectability is fundamentally governed by diffusion physics rather than raw temperature measurements.

## 1. Introduction

### 1.1. Structural Health Monitoring and the Need for Non-Destructive Diagnostics

The rising complexity of contemporary engineering structures, together with increasingly stringent demands on operational safety, durability, and sustainability, has greatly amplified the demand for sophisticated structural health monitoring (SHM) systems [1,2]. Aging infrastructure, environmental damage, repeated loading, corrosion, and extreme weather conditions can gradually undermine the structural integrity of buildings, bridges, tunnels, composite components, and industrial facilities [1,3,4,5,6,7,8,9,10]. As a result, the early identification of defects has become a key concern in civil engineering, materials science, and infrastructure management [11,12].

Structural health monitoring [13] aims to continuously or periodically assess the condition of structures using non-destructive testing (NDT) techniques capable of identifying damage without interrupting normal operation [14,15,16,17,18]. In reinforced concrete structures, typical defects include delamination, cracking, debonding, moisture accumulation, voids, honeycombing, and corrosion-induced degradation [19,20,21,22,23,24,25]. Undetected damage may lead not only to reduced mechanical performance and shortened service life, but also to increased maintenance costs, traffic disruption, and safety hazards [11,26,27].

Traditional NDT methods—such as ultrasonic testing, impact-echo, acoustic emission, and ground-penetrating radar—typically offer high sensitivity but often demand direct coupling to the surface, time-consuming scanning operations, or sophisticated data interpretation [28,29]. Against this backdrop, infrared thermography has gained prominence as a valuable complementary diagnostic tool, thanks to its capacity to quickly map spatial temperature fields linked to subsurface flaws and altered heat transfer behavior [30,31].

### 1.2. Infrared Thermography as an Emerging Diagnostic Tool

Infrared thermography (IRT) is a non-contact diagnostic technique based on the detection of infrared radiation emitted by the surface of an object [32]. Since the measured thermal response is directly related to heat transfer phenomena occurring within the material, thermography enables indirect identification of structural discontinuities, defects, and regions with altered thermophysical properties [18,33,34,35,36].

One of the key benefits of infrared thermography is its capability to quickly inspect extensive areas in a full-field manner, without the need for physical contact or disrupting normal operations [31]. In contrast to point-based diagnostic approaches, thermography delivers spatially continuous data on temperature distributions, which makes it especially well-suited for structural health monitoring of large-scale civil engineering assets [27]. Furthermore, current infrared camera systems exhibit high thermal sensitivity, allowing them to detect temperature variations often below 0.05 °C when measurement conditions are favorable [37,38].

Infrared thermography has been widely implemented across many engineering disciplines, such as reinforced concrete assessment, aerospace composite evaluation, photovoltaic system diagnostics, energy system analysis, additive manufacturing control, electronics cooling, cultural heritage conservation, and industrial process surveillance. Within civil engineering, thermography is frequently employed to identify subsurface delaminations, moisture-induced deterioration, cracking, debonding of strengthening materials, thermal bridging, and defects in insulation [2,12,27,39,40,41,42,43,44,45,46,47].

Depending on how thermal excitation is applied, infrared thermography can be divided into passive [48,49,50,51] and active [11,17,18,52,53,54,55,56,57,58,59,60] methods. In passive thermography, one relies on naturally occurring temperature differences caused by the environment or by the normal operation of a component. In contrast, active thermography uses deliberately applied, controlled thermal stimulation to increase defect visibility and improve the signal-to-noise ratio [61]. In both cases, performance is largely determined by heat transfer behavior, thermal diffusivity, defect shape and size, surface emissivity, and surrounding environmental conditions [20,33,34,51,52,62,63,64,65,66,67,68,69,70,71,72].

### 1.3. Current Research Challenges and Knowledge Gaps

Despite the rapid development of infrared thermography, several important challenges remain unresolved, particularly regarding the quantitative assessment of defect detectability in reinforced concrete structures. In many applications, thermographic interpretation still relies mainly on qualitative analysis of temperature distributions rather than standardized quantitative criteria. Consequently, the relationship between thermal contrast, defect geometry, excitation conditions, and actual detectability remains insufficiently understood [25,29,40,73].

Defect detectability in thermography is governed by coupled heat transfer mechanisms rather than temperature measurements alone. Parameters such as thermal diffusivity, diffusion length, excitation frequency, emissivity, spatial resolution, and thermal sensitivity directly influence the generation and attenuation of thermal contrast. In reinforced concrete, interpretation becomes especially difficult due to heterogeneous composition, moisture variability, environmental exposure, and low thermal diffusivity. Factors such as solar radiation, wind, emissivity variations, and non-uniform heating may distort thermal patterns and lead to false-positive or false-negative indications solar [44,64,68,74,75,76,77].

Additional limitations arise from the spatial and thermal resolution of infrared systems [76,78]. The detectability of small or deeply embedded defects depends not only on heat diffusion phenomena, but also on camera sensitivity (NETD), pixel resolution, acquisition distance, and signal-to-noise ratio [28,76,78,79,80]. Although artificial intelligence methods are increasingly explored for automated thermographic analysis, their practical implementation in SHM systems remains limited, particularly under real environmental conditions [12,13,37,81,82,83]. Therefore, a physics-based understanding of heat transfer, thermal-wave propagation, and thermal contrast evolution remains essential for reliable defect detectability assessment in thermographic structural health monitoring [1,2,13,41,49,84,85].

The logic of this review stems from the observation that while individual components of IRT and AI are well-documented, a quantitative synthesis linking physical detection limits (e.g., thermal diffusivity and resolution constraints) with automated diagnostic accuracy remains a significant knowledge gap. Existing surveys often treat AI as an autonomous image-processing tool, frequently neglecting the fact that defect detectability is primarily dictated by heat transfer physics. By systematically identifying these disciplinary silos through an AI-augmented literature retrieval strategy, this work provides a structured path to bridge the gap between theoretical heat conduction and real-world, automated structural health monitoring.

### 1.4. Aim and Scope of the Review

The primary aim of this review is to identify and analyze the physical mechanisms, operational conditions, and technological limitations governing defect detectability in infrared thermography-based structural health monitoring (SHM) systems. Particular emphasis is placed on the quantitative relationship between heat transfer phenomena, thermal-wave propagation, thermal diffusivity, and the formation of defect-induced thermal contrast in engineering materials, with a focus on reinforced concrete.

To ensure a comprehensive synthesis of the state-of-the-art, the literature retrieval strategy involved a systematic search of major academic databases, including Scopus, Web of Science, Google Scholar, and IEEE Xplore. The search was focused on key terms such as ‘infrared thermography’, ‘structural health monitoring’, ‘defect detectability’, ‘thermal diffusivity’, and ‘AI-assisted NDT’.

The logic of this review follows a progressive path from theoretical foundations to automated diagnostics. It begins by establishing the fundamental physics of infrared radiation and surface emissivity. It then transitions into the core mechanisms of transient heat conduction, highlighting how thermal diffusivity and effusivity govern the emergence of surface thermal patterns. This provides the basis for evaluating passive and active thermography techniques, their respective resolution limits, and penetration depths. The final stage of the review addresses the integration of these physical principles with signal processing and artificial intelligence (AI) to achieve automated and objective defect evaluation.

Unlike existing reviews that often focus primarily on either experimental case studies or the broad application of NDT methods, this work introduces a physics-oriented framework that explicitly links automated diagnostics to the fundamental limits of heat transfer. The novelty of this review lies in:

- Quantitative Detectability Criteria: Providing specific thresholds, such as the 0.5 °C minimum thermal contrast required for reliable detection.
- Analysis of Environmental Uncertainties: Quantifying the impact of moisture-induced emissivity variations (which can exceed 10%) on temperature measurement accuracy—a factor frequently overlooked in conventional reviews.
- Holistic AI Integration: Bridging the gap between classical heat diffusion theory and modern AI-assisted diagnostics (e.g., CNN, Transfer Learning), ensuring that automated models are interpreted within the context of physical detection limits.

By synthesizing these elements, the review provides a critical framework for the development of robust, intelligent SHM systems capable of operating under real-world environmental conditions.

To clearly delineate the novelty and specific contributions of this study compared to the existing literature, Table 1 summarizes the key differences between the proposed physics-oriented framework and conventional reviews in the field of infrared thermography and AI-assisted structural health monitoring.

While the title and primary focus of this review are dedicated to concrete structures, the manuscript deliberately incorporates a comparative analysis of other engineering materials, such as metals, polymers, and asphalt. This inclusion is grounded in a physics-oriented framework. By contrasting the low thermal diffusivity of concrete with the high conductivity of metals or the insulating properties of polymers, we establish the fundamental sensitivity and resolution limits of infrared thermography.

Furthermore, the detailed discussion on composite materials (FRP/CFRP) and nanotube-reinforced (CNT) matrices is essential, as these materials are increasingly integrated into concrete infrastructure as external strengthening systems or embedded smart sensors. Their inclusion allows for a holistic assessment of Structural Health Monitoring (SHM) for modern, hybrid concrete-composite systems, ensuring the review remains relevant to contemporary engineering practices.

## 2. Fundamentals of Infrared Thermography in Structural Health Monitoring

This section examines the physical mechanisms that determine how defects can be detected using infrared thermography, with a specific focus on pinpointing the parameters that constrain spatial resolution [76,78], penetration depth [42,72,86,87,88], and measurement accuracy [28,89].

Table 2 summarizes typical thermophysical properties of selected engineering materials relevant to infrared thermography together with their implications for defect detectability [57,72]. Materials with lower thermal diffusivity generally enable deeper defect detection due to slower heat propagation [90] and enhanced thermal contrast [41].

To provide a comprehensive understanding of the physical constraints governing infrared diagnostics, Table 2 summarizes the thermophysical properties of a broad range of engineering materials. While the primary focus of this study is reinforced concrete, the inclusion of metals, polymers, and advanced composites serves two critical purposes. First, it establishes a comparative baseline: by contrasting the low thermal diffusivity of concrete with the high conductivity of metals (e.g., aluminum, copper) or the insulating nature of polymers, we can more precisely delineate the sensitivity and resolution limits specific to concrete structures. Second, this broader scope reflects the reality of modern structural health monitoring, where concrete elements are frequently integrated with external strengthening systems, such as FRP/CFRP laminates, or repaired using epoxy-based resins. Understanding the thermal interaction between these diverse materials is essential for the reliable detection of debonding and interfacial defects in hybrid structural systems.

As shown in Table 2, materials with lower thermal diffusivity (e.g., polymers and composites) generally enable deeper defect detection due to slower heat propagation and enhanced thermal contrast, whereas highly conductive metals such as aluminum, steel, and copper require fast excitation techniques and careful emissivity control [51,65]. The rationale for creating this table stems from showing the parameters influencing the thermographic response of the material. To properly assess how a material behaves in infrared thermography, one must determine its thermophysical properties—namely, thermal conductivity (k), density (ρ), and specific heat capacity (c_p_)—which together define the thermal effusivity (e).

Materials with high thermal effusivity absorb heat more rapidly (e.g., metal), making them appear colder, even if they have the same temperature as a material with low effusivity (e.g., wood). In active pulsed thermography, effusivity allows the detection of subsurface defects (e.g., delaminations) because the defect area has a different effusivity than the homogeneous material, which the infrared camera records as a temperature difference. This coefficient is crucial when analyzing the dynamics of surface temperature changes, not just its steady state.

As shown in Table 2, both CFRP and GFRP composites display anisotropic thermal behavior. However, CFRP generally exhibits much higher in-plane thermal conductivity because of the inherently high conductivity of carbon fibers [95], while GFRP responds more like a thermally insulating material [96].

As it happens, adding carbon nanotubes (CNTs) to the polymer matrix exerts a transformative influence on these thermophysical interactions [94]. Owing to their outstanding thermal characteristics, even a small amount of CNTs markedly increases the effective thermal conductivity (k) and alters the specific heat capacity (cp) of the composite. These modifications directly affect the thermal effusivity (e), thereby boosting the material’s responsiveness to thermal excitation and improving the detectability of subsurface features during infrared inspections [97,98].

In practice, these changes in thermal effusivity lead directly to a stronger thermal contrast and an enhanced signal-to-noise ratio in infrared images. By speeding up surface heat dissipation and modifying the transient thermal behavior, the incorporation of CNTs enables more accurate detection of structural non-uniformities and potential defects that would otherwise go unnoticed in a conventional GFRP matrix [99].

It should be emphasized that although the study by [94] offers important findings on the mechanical enhancement of GFRP via CNT alignment, it does not examine the thermophysical properties of the resulting composite. Therefore, it is still necessary to investigate the thermal effusivity and radiative behavior of such nano-reinforced materials in order to fully harness their capabilities in advanced infrared diagnostic applications.

While this review focuses on concrete, the inclusion of nano-reinforced composites (CFRP/GFRP) in our analysis is essential because these materials are the primary media for modern concrete strengthening systems. The addition of carbon nanotubes (CNTs) serves as a case study in how targeted microstructural modifications can overcome the low thermal contrast typically associated with cementitious and polymer-based matrices.

**Figure 1:**
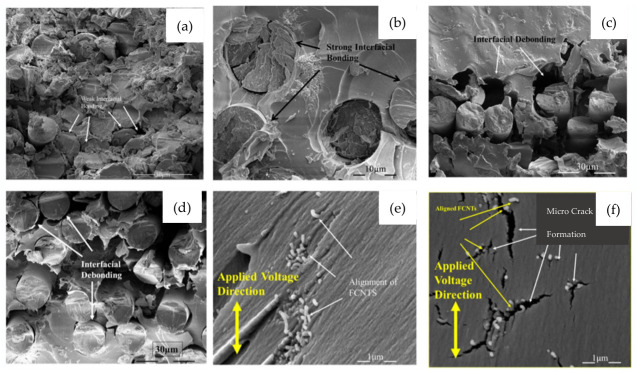
SEM images of GE and AFCNT-GE composites showing (**a**,**b**) the effect of CNTs on interfacial adhesion, (**c**,**d**) debonding caused by thermal loading, and (**e**,**f**) the orientation of FCNTs within the matrix. Reprinted with permission from [94], John Wiley and Sons.

Although the SEM micrographs in Figure 1 are primarily used to demonstrate the mechanical integrity and interfacial bonding of the composites, they simultaneously offer a structural framework for interpreting their thermal behavior. The progression from poor interfacial bonding in the GE composite (Figure 1a) to the robust interfacial adhesion in the AFCNT-GE composite (Figure 1b) is especially important, since a well-formed interface lowers thermal contact resistance. In addition, the FCNT alignment depicted in Figure 1e promotes more effective heat conduction pathways. These structural characteristics, which the authors mainly discuss in terms of mechanical strength and debonding (Figure 1c–f), are in fact the same features that govern the improved thermal conductivity (k) and altered effusivity (e) necessary for high-contrast infrared imaging.

As a result, the microstructural integrity shown in Figure 1 acts as a key indicator of the material’s thermal behavior and its detectability in infrared NDT (Non-Destructive Testing) applications.

The microstructural integrity captured in the SEM images (Figure 1) acts as a structural proxy for understanding heat transfer impedance. For instance, the transition from poor to robust interfacial bonding directly explains the reduction in thermal contact resistance, which is the same physical mechanism that allows IRT to distinguish between sound concrete and FRP-concrete debonding. Thus, these micrographs provide a fundamental physical explanation for the ‘hot spots’ observed during the inspection of reinforced infrastructure.

In conclusion, the results summarized in Table 2 indicate that optimizing the composite’s microstructure is the main factor responsible for its improved thermal contrast. The combined influence of strong interfacial adhesion and well-aligned CNTs produces a markedly higher signal-to-noise ratio, which is essential for dependable non-destructive evaluation (NDE) via infrared thermography.

Table 3 summarizes the critical correlation between the microstructural features observed in the SEM analysis and their direct impact on the thermophysical parameters governing infrared imaging.

### 2.1. Infrared Radiation and Emissivity of Materials

In concrete diagnostics, the practical application of Planck’s and Stefan-Boltzmann’s laws is primarily constrained by the uncertainty of surface emissivity (e). For cement-based materials, e is not a fixed property but varies with moisture levels and surface degradation [20,68]. Aging mechanisms such as carbonation, erosion, and the accumulation of contaminants typically increase surface roughness, which in turn tends to stabilize or slightly raise emissivity by promoting more diffuse radiation [66,100]. A 10% change in emissivity—common during the drying of concrete—can lead to significant temperature estimation errors, potentially masking the subtle 0.5 °C thermal contrast required to identify subsurface delaminations [101].

**Figure 2:**
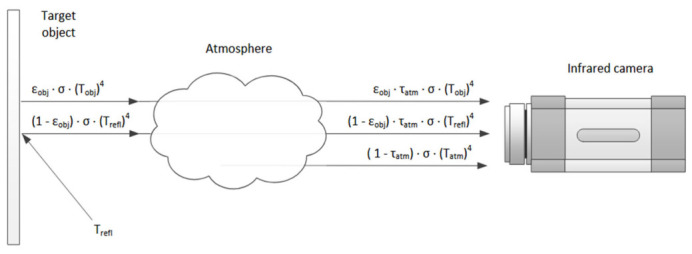
Radiation detected by the infrared camera (adopted from [14]).

In the literature, a schematic depiction of the radiation components captured by an infrared camera is frequently provided (for example in [14,28]). This model shows that the overall measured signal is composed of the object’s own emitted radiation, radiation from the surroundings that is reflected by the object, and contributions from the atmosphere as illustrated in Figure 2. Such a diagram is crucial for understanding measurement uncertainties and for developing appropriate calibration methods.

Reported emissivity values in the literature [102] show that building and polymer-based materials generally have high emissivity (e.g., concrete and asphalt ε ≈ 0.90–0.98, polymers ε ≈ 0.90–0.95) [51,76]. Fiber-reinforced composites such as CFRP and GFRP typically span ε ≈ 0.80–0.95, depending on their surface conditions [69,93]. In contrast, metallic materials exhibit much lower and more variable emissivity (e.g., steel ε ≈ 0.20–0.80, aluminum ε ≈ 0.05–0.10, copper ε ≈ 0.03–0.05) [65], which directly influences the accuracy of thermographic measurements.

**Figure 3:**
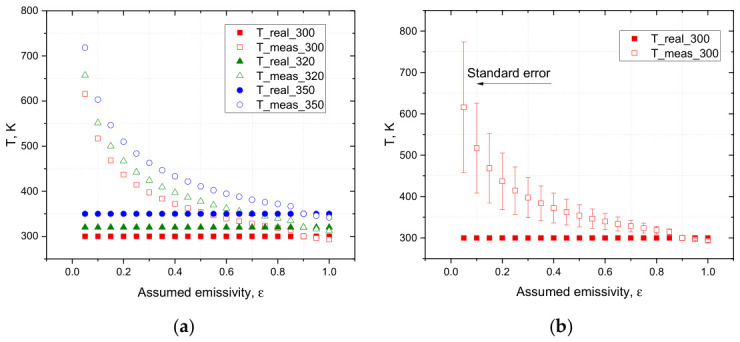
Effect of emissivity on temperature determination using the Stefan–Boltzmann law: (**a**) inferred temperature as a function of the assumed emissivity; (**b**) relative temperature deviation (on the basis of [10,103,104]).

The effect of emissivity on temperature measurement is commonly demonstrated using simplified models that depict how detected radiation changes with different surface properties. The analysis shown in Figure 3 presumes a fixed true emissivity (ε = 0.9) and illustrates how the error increases as the assumed emissivity departs from the actual value. These diagrams emphasize that inaccurate emissivity settings can cause significant temperature measurement errors, especially in industrial applications, building diagnostics and energy systems [10,103,104].

One of the primary parameters influencing the emissivity of concrete is its moisture content. Newly cast or damp concrete shows elevated emissivity values because of the water it contains, as water itself has a high emissivity (ε > 0.96). As the surface dries out, modifications in the microstructure and pore arrangement change the effective emissivity, causing the material’s radiative properties to vary over time.

**Figure 4:**
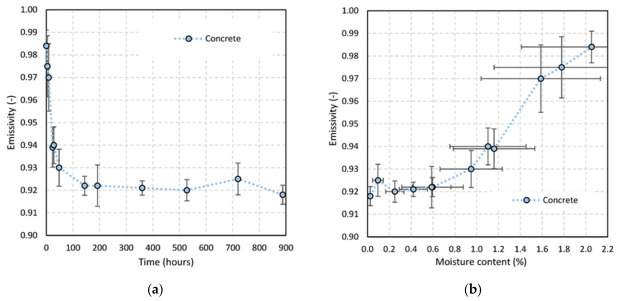
Emissivity of concrete versus time; (**a**) emissivity of dry concrete; (**b**) emissivity of moisture concrete (adapted from [64], CC BY 4.0).

As shown in Table 4 and in Figure 4, emissivity variations in concrete may reach several percent depending on surface condition, which can introduce non-negligible errors in thermographic measurements if not properly accounted for.

Moisture content has a significant impact on the emissivity of construction materials, including concrete (see Figure 4b) what was vividly discussed in [64]. Experimental studies conducted during controlled drying processes have shown that emissivity may vary by more than 10% depending on the moisture level. At high moisture content, emissivity values approach unity (ε ≈ 1.0), primarily due to the dominant radiative properties of water. As drying progresses (see Figure 4a), a gradual decrease in emissivity is observed, corresponding to the reduction in water content within the material. In the case of concrete, this decrease occurs immediately without a clearly defined initial stabilization phase, unlike some other porous materials. The emissivity evolution can be generally divided into three stages: an initial high-emissivity state, a transition phase characterized by decreasing emissivity and increased variability, and a final stabilization phase in which emissivity reaches a relatively constant value. The transition phase is particularly important from a measurement perspective, as it is associated with increased dispersion of emissivity values, reflecting the heterogeneous distribution of moisture within the material. Even relatively low moisture levels (on the order of 1.5% for concrete) are sufficient to induce measurable changes in emissivity, which may affect the accuracy of thermographic temperature estimation.

From a measurement standpoint, assuming a constant emissivity (e.g., ε = 0.95) can lead to systematic errors in temperature estimation when the true emissivity changes over time due to drying processes or environmental exposure [64]. This issue is particularly important in transient thermographic studies, where observed temperature variations may partly result from emissivity changes rather than actual thermal behavior. In addition, the effect of reflected radiation must be taken into account. While the relatively high emissivity of concrete diminishes the impact of reflected ambient radiation compared with low-emissivity materials (such as metals), this influence is still significant, especially in controlled laboratory settings or when high measurement precision is required. Changes in emissivity directly influence the reliability of defect detection, as they affect how thermal contrast is interpreted in infrared imaging [20,64,66,67,68,100,101].

Infrared thermography has found extensive use in numerous engineering fields, such as diagnosing building envelopes, evaluating the thermal performance of installations, and examining heat transfer phenomena. Research conducted by Wciślik and collaborators [10] has shown that thermography is suitable for assessing thermal conditions in building components, as well as in wider energy and heat transfer systems [104]. Their studies confirm that thermography is effective not only for locating flaws in construction materials but also for analyzing thermal behavior in engineering systems [28,35,62].

Moreover, thermographic techniques have been effectively applied across multiple areas of heat transfer analysis—such as energy systems, fluid flow diagnostics, and evaluations of thermal performance—demonstrating their flexibility and cross-disciplinary importance [102,105]. Combining thermography with quantitative heat transfer analysis provides deeper insight into thermal behavior and facilitates the development of advanced diagnostic approaches [71,106].

### 2.2. Heat Transfer Mechanisms Governing Thermographic Response

The thermographic response of concrete is governed primarily by transient heat transfer mechanisms, with heat conduction playing the dominant role [34,47,70,107]. Grasping how these mechanisms interact is crucial for properly interpreting thermographic data and for designing robust diagnostic techniques [63]. In numerous real-world scenarios, simplified models treat heat conduction as one-dimensional [50], yet actual systems frequently involve multidimensional heat transfer, especially in anisotropic materials like composites [108]. Experimental investigations using step heating thermography (SHT) have demonstrated that defect detectability is not directly related to absolute temperature values, but rather to disturbances in heat flow caused by subsurface discontinuities [106]. The heat transfer process in defective concrete can be divided into three characteristic stages during heating:

(i) simultaneous heat-up of both sound and defective regions,
(ii) development of lateral heat flow above the defect,
(iii) stabilization of maximum thermal contrast.

The second stage is particularly critical, as the onset of lateral heat flow generates a measurable surface temperature gradient, which enables defect detection by infrared thermography [41,50].

**Figure 5:**
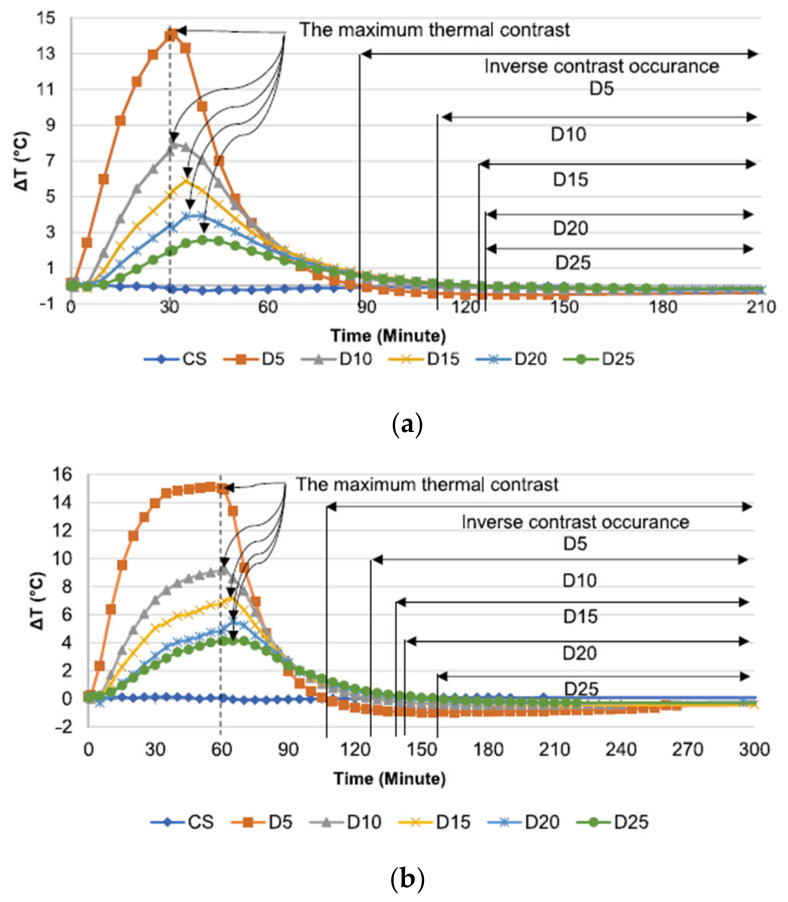
Evolution of surface temperature distribution during step heating thermography and the formation of thermal contrast above subsurface defects; the development of lateral heat flow for (**a**) thirty and (**b**) sixty minutes heating (adapted from [106]).

Quantitative results [106] indicate that the magnitude of thermal contrast strongly depends on defect depth. For shallow defects (5 mm), the maximum thermal contrast reaches approximately 14.1 °C, whereas for deeper defects (25 mm) it decreases to about 2.6 °C under comparable heating conditions. This represents a reduction of nearly fivefold, confirming the strong attenuation of the thermographic signal with depth. Furthermore, the influence of heating duration becomes more pronounced for deeper defects, where an increase in heating time may enhance thermal contrast by up to 57.7%, compared to only about 7% for shallow defects. During the cooling phase, the heat transfer mechanism reverses, leading to the formation of inverse thermal contrast, where the defective region becomes cooler than the surrounding material. The minimum inverse contrast values may reach approximately −0.98 °C, indicating a measurable but significantly weaker signal compared to the heating phase. These observations confirm that thermographic measurements should be interpreted in terms of heat transfer dynamics, particularly thermal diffusion and lateral heat flow, rather than surface temperature alone [39]. Consequently, the detectability of subsurface defects is controlled by the conditions required for the development of lateral heat flow and sufficient thermal contrast, typically exceeding 0.5 °C for reliable detection. The evolution of the thermographic response during both heating and cooling phases is illustrated in Figure 5a,b, highlighting the development of lateral heat flow, the formation of thermal contrast, and its subsequent inversion.

A solid grasp of the basic mechanisms of heat transfer is crucial for developing advanced infrared thermography–based damage detection methods [90]. Heat conduction, in particular, dictates how thermal disturbances spread within a material, while convection and radiation establish the boundary conditions that shape the observed surface temperature. Together, these processes control the emergence of thermal patterns linked to subsurface flaws, which represent the main information source in thermographic assessment. From the standpoint of intelligent damage detection, heat transfer physics defines the latent structure of the data recorded by infrared cameras. The spatial and temporal evolution of temperature fields embeds information on material characteristics, defect size and shape, as well as defect depth, which can then be leveraged through sophisticated data processing and machine learning algorithms. Consequently, a thorough knowledge of heat transfer behavior is a fundamental requirement for designing robust AI-supported diagnostic procedures [3,82]. Recent research underscores the increasing convergence of thermographic methods with intelligent data analysis within structural health monitoring applications [85,109]. In this framework, thermography functions not only as a non-destructive evaluation tool but also as a data-rich modality for automated defect identification, categorization, and predictive maintenance. As a result, integrating heat transfer modeling with artificial intelligence has emerged as a central research avenue consistent with the objectives of intelligent damage detection technologies.

The heat transfer mechanisms described above are quantitatively represented by the concept of thermal diffusivity, which is crucial for determining the detectability of defects.

### 2.3. Thermal Diffusivity and Defect-Induced Thermal Contrast

Thermal diffusivity, m [33,34] governs how quickly heat spreads within a material [110]. Materials with high thermal diffusivity (such as metals) dissipate heat rapidly, whereas those with low thermal diffusivity (such as polymers and composites) preserve temperature gradients for extended durations. Consequently, this property is essential in infrared thermography, as it directly affects how easily subsurface defects can be detected.

In pulsed thermography, the way the thermal contrast changes over time is closely linked to how deep a defect is located. Defects near the surface lead to an early and pronounced temperature contrast, while deeper defects result in weaker signals that emerge later. This behavior reflects the diffusion-driven mechanism of heat transfer and can be exploited to infer the depth of defects.

The size and progression of the thermal contrast are influenced by several factors:

- Defect depth and size [86];
- Thermal properties of the defect (e.g., air-filled void vs. inclusion) [28];
- Excitation conditions (e.g., energy input) [8];
- Thermal diffusivity of the material [110].

Materials with low thermal diffusivity generally produce stronger thermal contrast because heat spreads more slowly, giving defects a greater influence on the surrounding temperature field. Conversely, materials with high thermal diffusivity demand fast data acquisition, as heat dissipates quickly. The interplay between thermal diffusivity and the visibility of defects underpins the physical foundation of quantitative thermographic evaluation. By examining the time-dependent evolution of temperature distributions, one can extract information regarding defect depth and the thermal characteristics of the material. This concept is extensively employed in advanced thermographic methods such as pulsed and lock-in thermography, as well as in signal processing approaches like thermal signal reconstruction and phase-based analysis [39,51,111].

**Figure 6:**
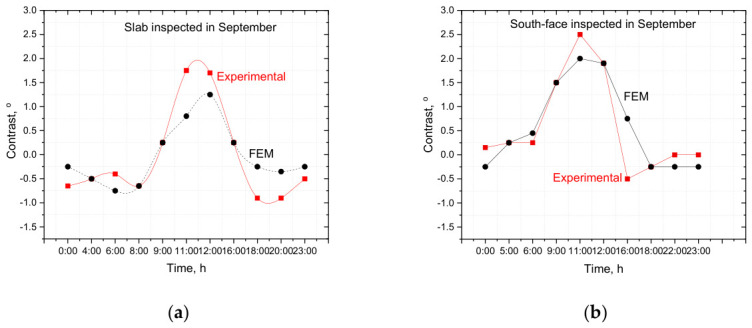
Temporal evolution of thermal contrast (ΔT) for defective and sound regions, comparing experimental measurements and FEM simulations, illustrating the diffusion-controlled nature of thermographic response and defect detectability for September (**a**) Slab, and (**b**) Column (adapted from [50]).

Thermal diffusivity governs the propagation of heat in concrete and directly controls the formation of defect-induced thermal contrast. The thermographic signal is defined as the temperature difference between defective and sound regions (ΔT = T_def_ − T_ref_), and its evolution is strongly time-dependent due to diffusive heat transport. Experimental and numerical results [50] indicate that reliable defect detection requires a minimum thermal contrast of approximately 0.5 °C, in accordance with ASTM guidelines. The Finite Element Method model reproduces this behavior with an uncertainty of about ±0.91 °C, confirming its adequacy for representing real heat transfer conditions. The magnitude of ΔT decreases with defect depth and lower size-to-depth (S/D) ratios, reflecting thermal attenuation during diffusion. Moreover, maximum contrast occurs within specific time windows during heating and cooling, indicating that detectability depends on both thermal diffusivity and transient boundary conditions [50]. The attenuation of thermal contrast with depth confirms that thermal diffusion length is the governing parameter defining the maximum detectable defect depth. The temporal evolution of thermal contrast for representative slab and column elements under different environmental conditions is presented in Figure 6, highlighting the agreement between experimental measurements and numerical predictions.

From the standpoint of intelligent damage detection, thermal contrast is the key feature derived from thermographic measurements. Its evolution in time and distribution in space can be further examined using machine learning methods, enabling automated detection and categorization of defects. Consequently, a solid grasp of thermal diffusivity and the mechanisms of contrast generation is crucial for linking conventional heat transfer theory with contemporary AI-supported diagnostic techniques. Recent studies on automated thermographic inspection of steel pipelines demonstrate that the temporal response of single pixels can be quantitatively utilized for defect assessment, including the determination of defect depth and thermal diffusivity. This shows that the diagnostic capability of active thermography resides not only in single-frame thermal images, but more importantly in the transient development of thermal contrast. In addition, enhanced defect detectability under low-contrast conditions underscores the significance of time-domain and physics-based analyses for robust defect characterization [112].

Experimental investigations into subsurface defect detection [80] demonstrate that flaws situated at depths between 0.1 and 0.4 mm, with diameters from 4 to 12 mm, can be consistently detected when measurement parameters are properly optimized. The findings also reveal that peak detection sensitivity is governed by both the defect shape and the specific measurement setup, with an ideal stand-off distance of about 1 mm providing stable and reproducible outcomes. In addition, the mismatch between numerical predictions and experimental optima underlines the influence of practical factors, including surface roughness and positioning precision.

Beyond geometric discontinuities, defects can strongly affect the inherent thermal behavior of materials [39]. The measured thermal diffusivity drops from 3.88 × 10^−5^ m^2^/s in the defect-free material to 3.84 × 10^−6^ in the defect-rich sample, corresponding to nearly an order-of-magnitude decrease. This pronounced reduction underscores the predominant influence of defect-driven phonon scattering in constraining heat transport.

Numerical calculations and simulation results of [113] show that the thermal contrast caused by defects develops over a characteristic time scale dictated by heat diffusion. For instance, a subsurface defect at a depth of 8 mm generates a strong thermal contrast after roughly 100 s, which corresponds to heat accumulating above the defect as a result of its thermal impedance. As the temperature field continues to evolve, this contrast slowly decreases and becomes insignificant after around 1100 s, marking the approach to thermal equilibrium. In addition, phase analysis reveals a maximum phase contrast of 34.2° at a frequency of about 2.5 mHz, confirming the high sensitivity of frequency-domain thermography to subsurface defects.

Quantitative studies in the literature [40] show that defect detectability is highly sensitive to both depth and lateral size. For example, the smallest defect diameter that can be reliably detected increases from roughly 3 mm at a depth of 3 mm to around 11.5 mm at a depth of 8 mm, clearly illustrating the strong constraint imposed by thermal diffusion. In composite materials, robust detection generally necessitates a diameter-to-depth ratio of no less than 3. In addition, thermographic methods can reach measurement uncertainties as low as 2–5% under optimal conditions, though this can rise to about 10% for defects that are small or have complex shapes. Overall, these results demonstrate that the generation and interpretation of thermal contrast are controlled jointly by material characteristics and measurement settings.

The numerical results presented here offer a quantitative foundation for determining optimal excitation parameters and reveal how thermal diffusion controls both the strength and the spatial profile of the measurable signal.

### 2.4. Limitations Related to Spatial and Thermal Resolution

**Figure 7:**
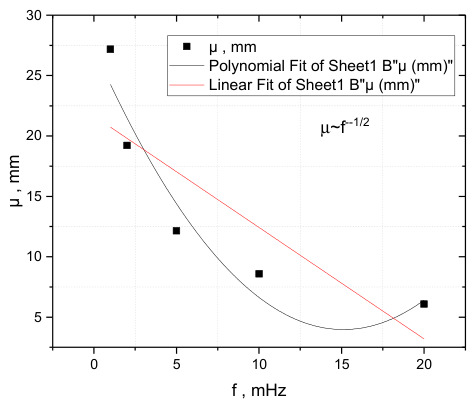
Thermal diffusion length (μ) as a function of excitation frequency (f); the typical inverse square-root dependence (μ~f^−1/2^). Based on data [112,113].

The spatial and thermal resolution achievable with thermal-wave diagnostic methods is intrinsically constrained by the diffusive character of heat transport. The principal parameter that dictates these constraints is the thermal diffusion length, given by μ = √(α/(πf)). As an example, the diffusion length drops from about 27.18 mm at 1 mHz to 6.08 mm at 20 mHz (see Figure 7), clearly demonstrating the inherent compromise between penetration depth and spatial resolution [57,114].

At low excitation frequencies, thermal waves can reach greater depths within the material, but this also causes pronounced lateral diffusion of the signal, which diminishes spatial resolution. Conversely, using higher frequencies enhances spatial resolution while restricting the effective inspection depth. As a result, shallow defects (e.g., h ≈ 3 mm) yield steep phase gradients and high spatial resolution, whereas deeper defects are associated with larger phase delays but much lower lateral contrast [113].

**Figure 8:**
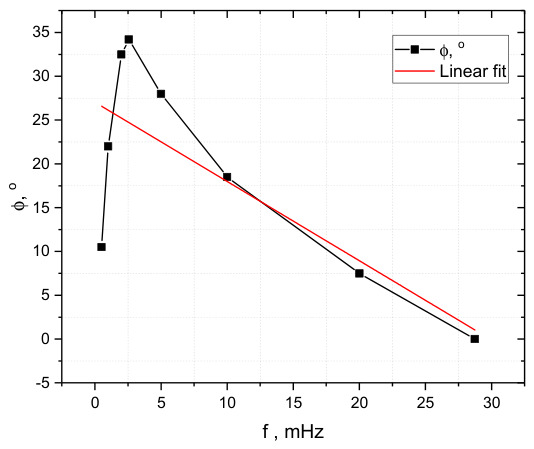
Phase difference (Δφ) as a function of excitation frequency (f). Based on data [112,113].

The maximum phase contrast is also frequency-dependent, reaching values of approximately 34.2° at an optimal excitation frequency of about 2.56 mHz (see Figure 8). Beyond this point, the phase contrast decreases, and a so-called blind frequency (~28.74 mHz) may occur, at which defect detection becomes ineffective.

Another important limitation concerns the size and depth of defects. Experimental results show that the smallest detectable defect diameter increases from roughly 3 mm at a depth of 3 mm to around 11.5 mm at a depth of 8 mm, clearly illustrating the strong attenuation of thermal signals with increasing depth [40]. This phenomenon imposes a fundamental limit on the spatial resolution achievable in subsurface defect detection. In real-world scenarios, measurement accuracy is further constrained by experimental conditions: typical errors range from 2.2% to 4.9% under well-controlled setups, but can rise to about 10% for smaller or more complex defects [40]. Taken together, these constraints demonstrate that attainable spatial and thermal resolution is determined by a complex interaction among excitation frequency, material characteristics, defect geometry, and signal processing settings. Consequently, optimizing the measurement configuration is crucial to achieve an appropriate compromise between penetration depth and resolution.

As the frequency increases from 1 mHz to 20 mHz (see Figure 7), the diffusion length decreases from 27.18 mm to 6.08 mm, revealing a pronounced reduction in the depth of thermal penetration. This dependence imposes a fundamental constraint on thermal-wave methods: gaining higher spatial resolution at elevated frequencies inevitably comes at the cost of a shallower probing depth.

In the study [115], infrared thermography was implemented using an unmanned aerial vehicle (UAV) to detect subsurface defects in concrete bridge decks under field conditions, providing a practical assessment of method limitations related to spatial and thermal resolution. From the perspective of spatial resolution, the results demonstrate that defect detectability is strongly governed by the ground sampling distance (GSD), which increases with flight altitude according to:

(1) GSD≈H·IFOV,

where H is the measurement height and IFOV is the instantaneous field of view (angular resolution) of the detector. An increase in H therefore directly leads to a degradation of spatial resolution and the occurrence of signal averaging (mixed pixel effect).

For typical UAV configurations, GSD values on the order of ~2–5 cm/pixel were reported, implying that defects smaller than approximately 2–3 pixels (≈5–10 cm) cannot be reliably resolved due to signal averaging (mixed pixel effect). Regarding thermal resolution, the study indicates that detectable temperature contrasts between defective and sound were relatively low (ΔT ≈ 1–3 °C), making defect identification highly sensitive to environmental conditions such as solar loading and convective heat losses. Under such conditions, the signal-to-noise ratio approaches the sensitivity limits of the infrared camera, reducing detection reliability. Furthermore, the study confirms that defect depth constitutes a fundamental limitation, as the thermal response amplitude decreases with depth due to diffusive heat transport, which can be approximated by the characteristic thermal diffusion length μ. Consequently, deeper defects generate significantly attenuated surface signals, often below the detection threshold. Overall, the results highlight that, in real-world applications, defect detectability does not follow a fixed efficiency metric but depends on the combined influence of sensor parameters (IFOV, NETD), measurement geometry (UAV altitude), and environmental conditions, leading to reduced detection capability for small, low-contrast, or deeply embedded defects.

These constraints establish the practical range within which thermographic measurements can be carried out and must be taken into account when developing defect detection strategies for real-world applications.

### 2.5. Implications for Defect Detectability

The practical boundaries of defect detectability in concrete are not absolute but result from a complex interplay of the parameters described in Section 2.1, Section 2.2, Section 2.3 and Section 2.4. Specifically, the 0.5 °C thermal contrast criterion should be understood as a signal-to-noise threshold. Its reliability depends on the camera’s NETD and surface emissivity stability; as shown in Section 2.1, moisture-induced emissivity changes can exceed 10%, potentially masking this contrast. Similarly, the 5–10 cm detectable depth range is a diffusion-limited boundary. While passive methods often fail beyond 2–5 cm, reaching the 10 cm limit requires active excitation (e.g., step heating) and a favorable defect size-to-depth (S/D) ratio, typically ≥3. Consequently, detectability must be interpreted as a probabilistic quantity, where the likelihood of detection decreases as thermal signals attenuate with depth and environmental noise increases.

Having established the physical boundaries and resolution limits of IRT, the following section evaluates how different active and passive methodologies can be optimized to operate within these constraints.

## 3. Active and Passive Thermography Techniques

The limitations discussed in Section 2 highlight that defect detectability in infrared thermography is strongly dependent on thermal contrast generation and measurement conditions [41,50,71,106,116]. Consequently, different thermographic techniques have been developed to enhance or control the thermal response of a structure [41,50,71,106,116], which can be broadly classified into active and passive approaches.

The choice between passive and active thermography is dictated by the specific requirements of the structural inspection and the physical nature of the anticipated defects. To provide a systematic decision-making framework, Table 5 summarizes the key operational differences between these two approaches across seven critical criteria.

As summarized in Table 5, while passive thermography offers unparalleled scalability for infrastructure such as bridge decks, its reliability is often compromised by environmental noise that can lead to false-positive indications, such as oil stains misinterpreted as delaminations. Conversely, active thermography bridges the detection gap by providing the high contrast and depth control necessary for quantitative defect characterization, provided that the non-uniformity of the artificial heating is carefully managed.

**Figure 9:**
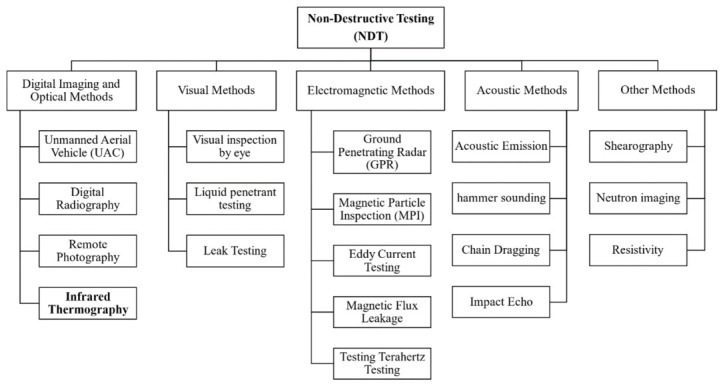
Overview and classification of non-destructive testing (NDT) methods. Reprinted from [31].

To place infrared thermography within the broader context of non-destructive testing (NDT), Figure 9 presents a general classification of inspection methods, highlighting its position among digital imaging and optical techniques. It illustrates the main categories of inspection techniques and the position of infrared thermography within digital imaging and optical methods [31].

### 3.1. Passive Thermography

Passive thermography is based on the observation of naturally occurring temperature differences on the surface of a structure, without the application of any external thermal excitation [119]. In this approach, thermal contrasts arise from environmental influences such as solar radiation, ambient temperature variations, wind, or internal heat generation during normal operation [120]. As a result, passive thermography is particularly well suited for large-scale, in situ inspections, including buildings, bridge decks, and photovoltaic systems [116].

From the perspective of defect detectability, as discussed in Section 2.5, the effectiveness of passive thermography is primarily governed by the availability and magnitude of naturally induced thermal gradients. Since no external excitation is applied, the achievable temperature contrast (ΔT) is typically limited, often remaining within a range of approximately 0.5–2 °C [50]. Consequently, the signal-to-noise ratio may be insufficient for reliable detection of small or deeply embedded defects, especially when the contrast approaches the sensitivity limit of the infrared detector. According to ASTM recommendations, a minimum thermal contrast of approximately 0.5 °C is generally required for reliable detection of subsurface delaminations in concrete structures [119].

In passive thermography, the thermal response is strongly dependent on transient environmental heat transfer conditions [48,50]. During daytime solar heating, defective regions often exhibit higher surface temperatures due to locally disturbed heat conduction and reduced heat dissipation. In contrast, during nighttime cooling, the thermal contrast may reverse, causing damaged areas to appear cooler than the surrounding sound material. This inversion phenomenon confirms that the detectability of defects is governed not by absolute temperature itself, but by the direction and dynamics of heat flow within the structure. In [50] it is illustrated the principle of passive thermography under daytime and nighttime conditions, highlighting the evolution and inversion of thermal contrast caused by natural heating and cooling cycles.

Moreover, passive thermography is highly sensitive to environmental conditions [76]. Variations in solar loading, convective heat transfer, cloud coverage, wind speed, and ambient temperature fluctuations can significantly influence the thermal response of the structure, leading to non-uniform heating and temporal variability of the signal. This introduces uncertainty in the interpretation of thermograms and may result in both false-positive and false-negative indications. In particular, changes in surface emissivity, moisture content, or reflections from surrounding objects may further distort the measured temperature distribution. In [50] it is presented the temporal evolution of thermal contrast for representative concrete elements under varying environmental conditions, demonstrating the agreement between experimental measurements and FEM predictions as well as the strong dependence of ΔT on daily heat flux variations.

Regarding heat transfer mechanisms, the thermal behavior observed in passive thermography results from the combined effects of heat conduction inside the material and convective–radiative exchanges at its surface. This behavior can be modeled using transient heat transfer equations that merge Fourier’s law of conduction with convective coupling and radiative energy balance. Because there is no controlled external excitation, the inspectable defect depth cannot be actively tailored and is instead constrained by the natural penetration of thermal disturbances, which depends strongly on the material’s thermal diffusivity and the defect’s size-to-depth (S/D) ratio. Both experimental measurements and numerical simulations show that defects located deeper within the material generate much weaker thermal contrast, owing to attenuation of the thermal signal as it diffuses.

Despite these drawbacks, passive thermography still presents notable benefits, such as low cost, ease of use, and the capability to quickly survey extensive areas without disturbing normal operations. As a result, it is frequently employed as an initial diagnostic method to pinpoint regions of interest that may need more detailed examination with more controlled methods, such as active thermography.

### 3.2. Active Thermography Techniques

Active thermography relies on the application of a well-controlled external heat stimulus to induce transient heat flow within the material under inspection. Unlike passive thermography, in which the thermal response is governed by ambient conditions, active thermography permits accurate adjustment of excitation parameters, thereby greatly enhancing the detectability of subsurface flaws. This method is especially relevant for reinforced concrete structures, where low thermal conductivity and high thermal inertia hinder the formation of thermal gradients that are sufficiently pronounced to be detected under natural conditions.

From a structural health monitoring standpoint, active thermography is particularly well suited to examine near-surface defects situated between the exposed concrete face and depths of roughly 10 cm. In this way, it effectively closes the detection gap between optical surface inspections and deeper non-destructive testing (NDT) methods such as ground-penetrating radar or ultrasonic testing [28]. The technique has been successfully used to identify delaminations, voids, honeycombed areas, cracks, CFRP laminate debonding, moisture-induced deterioration, and inadequately grouted prestressing ducts in concrete elements.

**Figure 10:**
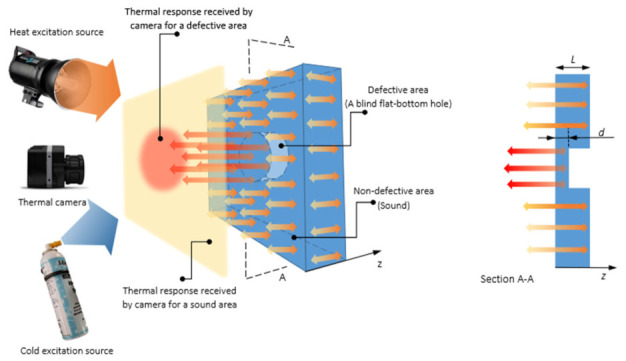
Schematic representation of heat diffusion above a subsurface defect during active thermographic inspection (adapted from [86]).

The principle of active thermography is based on transient heat diffusion triggered by an external energy source, such as flash lamps, halogen emitters, quartz radiators, lasers, hot air devices, microwaves, or ultrasonic excitation (see Figure 10) [53,86]. Once the material is thermally stimulated, heat spreads through it following Fourier’s law of heat conduction. Defects disrupt the local heat flow because of variations in thermal conductivity, density, specific heat, or interfacial thermal resistance, leading to detectable temperature differences at the surface that are captured by an infrared camera. 

**Figure 11:**
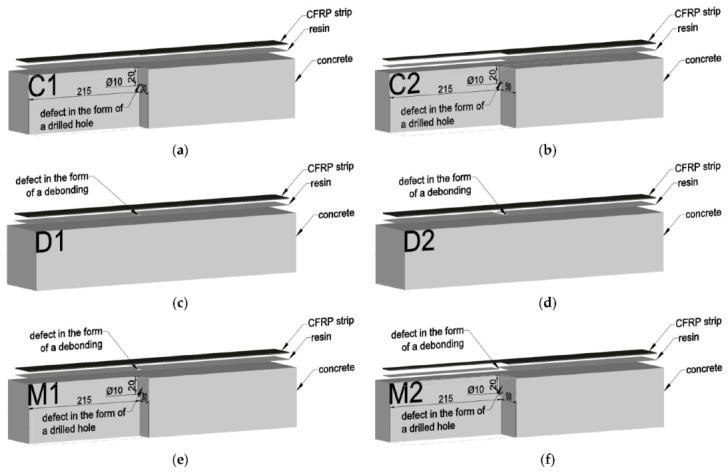
Location of defects in beams: (**a**,**b**) defects in the form of a hole under the composite–concrete defects, (**c**,**d**) defect in the form of debonding–bond defect in the adhesive layer, (**e**,**f**), samples with both types of damage—units are given in [mm] [28].

The performance of active thermography depends on how the excitation parameters, thermal diffusivity, and defect geometry interact. As outlined earlier, the thermal diffusion length dictates how deeply thermal waves can penetrate and can be adjusted by modifying the excitation frequency or the heating time. Thus, active thermography allows for limited tuning of inspection depth and spatial resolution based on the material under examination and the anticipated defect position, what is vividly discussed in [28] and can be presented as in Figure 11.

Several excitation strategies are currently employed in active thermography. Among them, pulse thermography (PT), pulse phase thermography (PPT), lock-in thermography (LT), and step heating thermography are the most widely used approaches for concrete structures.

#### 3.2.1. Pulse Thermography (PT)

PT involves applying a short, high-energy thermal pulse (ranging from milliseconds to several seconds) using flash lamps or halogen emitters. The key selection principle is balancing the pulse duration; while short pulses are necessary for high-resolution near-surface imaging, concrete’s high thermal inertia often requires longer pulses to deliver sufficient energy for deeper penetration.

This method is primarily effective for the rapid screening of near-surface defects (depth < 2–3 cm). It is the preferred technique for identifying shallow delaminations or air voids within the adhesive layers of externally bonded CFRP strengthening strips.

#### 3.2.2. Pulse Phase Thermography (PPT)

PPT transforms transient thermal signals into the frequency domain using Fast Fourier Transform (FFT). The parameter selection principle relies on analyzing phase images at specific frequencies; for concrete, low modulation frequencies (approximately 0.14 mHz) must be prioritized because higher frequencies are rapidly attenuated near the surface.

PPT is highly effective for structures with non-uniform surface emissivity or uneven heating, as phase images are significantly less sensitive to these artifacts than raw thermograms. It is particularly suitable for the quantitative lateral characterization of deep delaminations in bridge decks [54,110].

#### 3.2.3. Lock-In Thermography (LT)

This technique uses periodically modulated thermal excitation. The primary selection parameter is the modulation frequency (f), which is tuned based on the target inspection depth and material diffusivity. Due to concrete’s slow thermal response, very long heating periods—ranging from 10 to 60 min (frequencies between 1.67 mHz and 0.28 mHz)—are required for effective wave penetration.

LT is best suited for precise depth estimation of delaminations. While it offers high sensitivity up to 5 cm, it reaches a practical detectability limit in concrete at approximately 10 cm, where the phase delay typically becomes indistinguishable from environmental noise [54].

#### 3.2.4. Step Heating Thermography (SHT)

SHT involves long-duration, low-power thermal excitation. The critical parameter is the heating duration (th); for large-scale concrete elements, excitation times exceeding 1.5 h are often necessary to generate a measurable surface temperature gradient above deep-seated anomalies [54].

This is the most effective method for deep infrastructure diagnostics, such as detecting non-grouted regions in prestressing tendon ducts. It is also the preferred choice for damp concrete, as the prolonged heating duration helps overcome the increased thermal inertia caused by high moisture content [19,105].

In summary, active thermographic techniques provide the high thermal contrast and controllability necessary for quantitative Structural Health Monitoring (SHM). The integration of these physics-based methods with Artificial Intelligence (e.g., CNNs and Transfer Learning) further enhances their reliability by enabling automated defect categorization and the interpretation of low-contrast data. This synergy between heat transfer principles and advanced data processing represents the future of intelligent infrastructure diagnostics.

While the techniques discussed provide the necessary thermal data, their practical effectiveness in infrastructure health monitoring depends on the specific nature of the defect, as analyzed in Section 4.

## 4. Practical Applications in Concrete Structures

In addition to classic thermal imaging studies, which identify areas of heat loss to optimize heating/cooling costs, thermal imaging is widely used in non-destructive structural evaluation. This type of research is based on the relationship between surface temperature distributions of a structural element and the internal structure of the object, for example, the presence of voids or cracks.

This section describes examples of the use of thermography in the diagnosis of reinforced concrete structures, with particular emphasis on the detection of subsurface defects (voids, cracks, delaminations, etc.) and the identification of various destructive phenomena typical of concrete.

Basing on the theoretical issues discussed above, a literature review on the use of thermography in laboratory and full-scale testing is presented. Particular attention is paid to identifying the types of defects that can be identified using this technique. Additionally, optimal measurement conditions that allow for obtaining qualitative results are presented.

The limitations of the method are also discussed, primarily the sensitivity to environmental conditions and the limited depth range.

By synthesizing findings from prior studies, this section aims to provide a critical overview of the capabilities and constraints of thermography as a non-destructive evaluation tool for concrete infrastructure.

### 4.1. Defects Identification and Localization

IRT (especially in passive regime) is widely used in the diagnosis of concrete bridges. In recent years, the use of cameras mounted on drones has enabled very rapid data acquisition without restricting road traffic [61,121]. Another major advantage of thermal imaging is the ease of monitoring tall structures (e.g., chimneys, cooling towers) without the need for scaffolding or dangerous work at heights [70].

**Figure 12:**
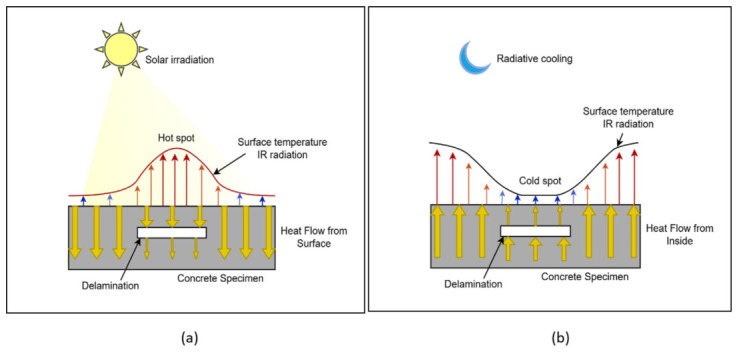
Principle of concrete damage detection with passive IRT: (**a**) Heating regime (day); (**b**) cooling regime (night), reprinted from [82].

A defect most frequently detected using IRT (primarily passive) is delamination (separation) of bridge deck slabs [9,49]. Detection of delamination at the concrete-steel pipe interface in Concrete-Filled Steel Tubes (CFST) is also possible [41]. Physically, delamination is a thin gap in the concrete element, filled with air or water. This layer acts as a barrier to heat transfer, but its effect varies depending on the ambient conditions [122]. During heating (at day), delamination hinders heat migration into the element, resulting in an increased concrete surface temperature in the delamination areas. Conversely, during cooling (at night), the void hinders heat transfer to the environment, which is visible in the thermographic image as an area with a lower surface temperature [31,82] (Figure 12).

**Figure 13:**
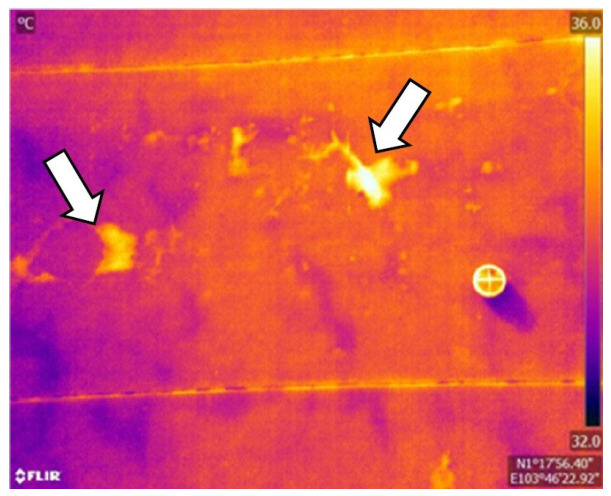
An example of concrete wall delamination detection using thermography (heating regime), from [4].

An example of a thermogram (heating regime), with visible delamination areas, is illustrated in Figure 13.

Filling a void/crack/delamination with water, for example, after heavy rainfall, reduces the ability of IRT to detect it, as water is characterized by a much higher thermal conductivity and specific heat than air.

The problem of assessing delamination in bridges using IRT is addressed in standard regulations, such as ASTM D4788-03 [119], which specify, among other problems, measurement conditions and guidelines for result interpretation. Particular attention is paid to determining the optimal time for conducting measurements. The standard [119] recommends that data collection should be conducted approximately 4–6 h after sunrise. This issue is discussed more extensively in the literature, where the optimal heating/cooling time is related, for example, to the depth of the defect location [44]. In most cases, however, authors agree that measurements conducted around noon yield the best results.

On the other hand, Kee et al. [43] found that sequential measurements conducted at night (bridge deck cooling) gave better results in detecting delamination.

Time-series data analysis is highly effective in detecting delamination [4]. Observing temperature changes over time provides new information compared to a single thermogram, as it allows for tracking the dynamics of heating/cooling phenomena. Delamination areas generally react more slowly than sound concrete. Another important advantage of this method is its low sensitivity to momentary disturbances (shade, wind), while simultaneously improving the accuracy of detecting small defects located at greater depths. The undoubted disadvantages of time-series analysis include long measurement time at stable conditions and the generation of large amounts of data for processing. This method is generally combined with active thermography, making it unsuitable for examining large surfaces.

Although the IRT method has great potential for delamination detection, performing measurements and obtaining qualitative results in open spaces (as in the case of bridges) is difficult and subject to many interferences. Therefore, it is recommended to supplement IRT results with other diagnostic methods, such as ground-penetrating radar (GPR), visual inspections, etc. [123]. A separate challenge is developing algorithms that optimally integrate data from various diagnostic methods [124]. Combining IRT and GPR is a particularly promising procedure [56], as both methods provide different information and operate on different geometric areas. IRT is predestined for effective detection of near-surface defects over large areas, while GPR is more effective in detecting deeper objects. Synthesis of the results obtained from both methods, due to the synergistic effect, provides a more comprehensive understanding of the structure of the examined object.

In the case of active thermography tests, obtaining high temperature contrast of sound and defective concrete is favored by high air humidity and high ambient temperature [59]. Tests performed in a thermal chamber, with strict control of the heating and cooling process, showed that the thermal contrast is linearly dependent on the initial temperature [71].

Paper [8] also addresses the issue of the influence of environmental factors (primarily external temperature) on measurement resolution. The differences between the defective and sound areas did not exceed 1–2 °C. Furthermore, it was observed that during the heating (from approximately 9 a.m.), the thermal contrast is positive, meaning that the area above the void is characterized by a higher temperature than the surrounding area. In contrast, during the evening and night (cooling regime), a negative contrast is observed (lower temperature above the void). As noted, around 8 a.m. and 6 p.m., the contrast is close to 0 (transition between heating and cooling regimes), indicating the inability to detect voids at that time. On the contrary, the greatest contrast, and therefore the best detection capabilities, were observed around noon. These conclusions are confirmed by analysis of thermograms recorded at 12 a.m., 5 a.m., and 8 a.m.

A literature review on defect detection in concrete bridges is presented in [101]. The authors paid particular attention to external factors that interfere with the measurement process and the interpretation of results. These factors are divided into three basic groups: environmental, physical, and technical. In field tests, environmental factors such as ambient temperature and changes in solar radiation intensity throughout the day are generally decisive. Cloud cover also results in variable heating conditions of the concrete surface, which can also affect the quality of the obtained data and the accuracy of their interpretation. When analyzing thermographic data, factors such as wind (faster surface cooling), concrete moisture, heat reflection from adjacent objects, and the obscuration of the analyzed surface by these objects should also be considered. As can be seen, the interpretation of results is subject to numerous interferences, particularly significant in passive thermography.

As demonstrated in [125], the recording and interpretation of accurate data can also be hampered by the varying condition of the concrete surface, which locally changes its emissivity. This applies to both the presence of contaminants and protective coatings, as well as the surface structure of the concrete itself. In general, rough surfaces are characterized by higher emissivity than smooth ones, which affects the measurement results, especially with subtle temperature differences [49].

In the case of bridges, the impact of oil stains and other petroleum-derived substances on the bridge deck surface is particularly significant. This locally changes the surface’s emissivity, which can be misinterpreted in a thermogram as a defect [126].

The ability to detect voids and delaminations using IRT depends on the depth of the defect location. As noted in [8], under favorable weather conditions, thermography was a very effective tool for detecting near-surface defects. For greater depths, ground-penetrating radar (GPR) and ultrasonic pulse echo (UPE) were more effective. In terms of assessing the shape and size of defects, IRT and GPR were similarly effective.

It is generally believed that IRT can effectively detect defects at depth of the order of several centimeters [127]. This problem was also observed in [128], where it was found that IRT images do not allow for the detection of small voids located at a depth greater than their lateral dimension.

The last issue was partly solved in [42]. Based on the results obtained in two regimes of the active procedure (heating the concrete surface from the outside), an equation relating the product of thermal diffusivity of concrete and observation time with the depth of the subsurface defects was determined. However, the authors were unable to locate defects deeper than 5 cm.

In [48], attention was drawn to the relationship between the defect size-to-depth (S/D) ratio and the ability to detect defects using passive thermography under various solar heating conditions. It was found that the S/D threshold at which defect detection is possible depends on the position of the examined surface relative to the direction of sunlight. As the authors noted, data postprocessing, primarily involving noise removal, improved the quality of the results by between ten and over 50%.

A comprehensive analysis of the effect of defect size and its depth position on the ability of active IRT to detect it was performed in [23]. By analyzing various configurations of 51 artificially introduced defects (based on own research and literature analysis), it was observed that thermal contrast analysis enables the detection of defects whose depth is smaller than or equal to their lateral dimension.

Large thickness of defects also promotes high thermal contrast and easy detection [61].

The temperature gradient on the concrete surface can also be used to detect cracks and micro-cracks. For example, Gu et al. [88] described a procedure that allowed for effective crack detection in a concrete tunnel structure. Passive thermography was used in the study, based on the natural temperature differences between the surrounding rock and the tunnel interior.

However, at small crack widths, the temperature difference between the defect and the surrounding concrete surface quickly disappears, making the detection of narrow cracks difficult. This limitation can be reduced, for example, by coating the concrete surface with a chemical reagent at a temperature higher than the ambient temperature, which, by penetrating the crack, generates the desired thermal contrast [129].

A separate area of IRT applications for detecting internal defects in concrete includes localization of thermal changes that accompany the development of cracks under cyclic [73] and dynamic [47] loads.

Thermography is also used to detect grouting faults inside tendon ducts. However, since these elements are usually located deep in the concrete, the active thermography method, in which heating is conducted for a long time, is effective in this case [16].

In summary, among all the damage mechanisms analyzed, delaminations and large air voids located near the concrete surface are the most likely to be detected by IRT. This is due to the high thermal conductivity contrast between air and concrete. Even under passive thermography conditions, these defects generate distinct thermal contrasts, either positive or negative, depending on the heating/cooling phase. The studies presented above demonstrate that thermography is an effective tool for detecting large, shallow discontinuities, primarily delaminations in bridge slabs and reinforced concrete walls. Detection efficiency decreases with the depth of the defect, increasing humidity, and decreasing the defect size-to-depth ratio.

Detecting cracks and micro-cracks in concrete is more problematic and uncertain, as thermal contrast quickly fades. The ability to detect cracks depends primarily on their width, environmental conditions, and the presence of moisture. The effectiveness of passive thermography in these cases is very limited and primarily limits itself to detecting wide cracks in the presence of moisture. Active thermography, however, offers much broader prospects, especially when combined with the use of chemical reagents that penetrate the crack.

### 4.2. Rebar Detection in Concrete

IRT can be used to detect and determine the distribution of reinforcing bars in concrete [130]. It should be emphasized that IRT does not detect the reinforcement itself, but only records the heat flow disturbances caused by its presence. The observed thermal anomalies depend on the thermal properties of the steel and concrete, as well as the geometry of the element and its heating conditions. In practice, the measurement may be further distorted by concrete moisture or the presence of subsurface voids.

Moreover, a limitation in this case is the depth of measurement. This makes detection using basic passive thermography difficult and unreliable. For this reason, active IRT is most often used, in which the reinforcing bars are heated without contact.

**Figure 14:**
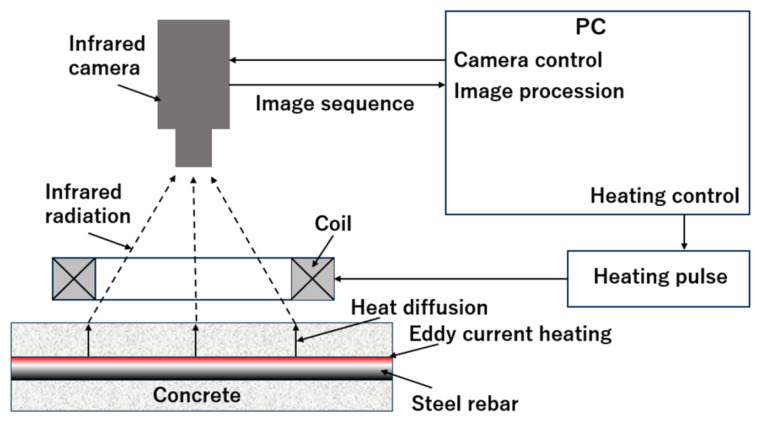
The use of active thermography for the reinforcement detection, from [24].

The most commonly described methodology involves active thermography, which uses the Joule effect in an electrical conductor to heat a rebar to achieve a sufficient temperature gradient (Figure 14). For this purpose, a coil is placed over the concrete element. After connecting the coil to an alternating current supply, eddy currents are generated in the reinforcement. The electrical resistance of the rebar causes its local heating. The heat thus generated propagates further towards the concrete surface and subsequently is recorded by the IRT camera.

In [131], a microwave excitation system was used to heat the bars, allowing for the smooth detection of bars located at a depth of up to 3.8 cm below the concrete surface. In most cases, this value is sufficient for structural diagnostics, but the suitability of this method for assessing, for example, earthworks, retaining structures, and foundations, where the concrete cover thickness is generally greater, may prove problematic.

The interpretation of thermograms and the identification of reinforcing bars can be further supported by deep learning tools, with the possibility of training them using the results of numerical simulations [55].

In general, the superficial nature of thermal imaging observations, combined with the presence of environmental disturbances, makes IRT the most commonly used technique for preliminary assessment of shallow-lying bars. When detailed inventory of reinforcement, especially deeper-lying, is required, the eddy current technique [60] or the ground-penetrating radar (GPR) [132] yield better results.

In summary, the depth of rebar position combined with its high thermal conductivity limits the usefulness of passive thermography for bar detection. Active thermography, which utilizes electromagnetic and microwave excitation, achieves significantly better results, especially for bars located at shallow depths. However, this method does not provide satisfactory results for thicker concrete covers, where GPR and eddy current techniques are more effective.

### 4.3. Corrosion Identification in Reinforced Concrete (RC) Structures

Corrosion of reinforcement in concrete structures is one of the primary phenomena that leads to their degradation and shortened service life. Visual detection of corrosion is only possible when it is significantly advanced, when the structure’s usefulness is already severely limited.

For this reason, in recent years, intensive research has been carried out on the possibilities of detecting corrosion initiation using semi-destructive and non-destructive testing (NDT) methods. The most commonly used methods include electrochemical, ultrasonic, X-ray tomography, ground-penetrating radar, magnetic flux leakage, as well as eddy current testing, and others [24].

In recent years, infrared thermography has emerged as a promising non-destructive technique for identifying areas affected by corrosion [121], owing to its ability to capture surface temperature variations associated with subsurface anomalies.

In IRT corrosion diagnostics, it is important to distinguish between the actual diagnostic goal and the physical source of the observed thermal anomalies. Thermography does not record the corrosion products themselves, but rather the presence of the phenomena that accompany them, primarily the formation of microcracks, as well as the migration of moisture and salts. Consequently, thermal responses are generated by various phenomena accompanying corrosion.

Corrosion products located on the bar surface are characterized by different thermal properties than the base metal, particularly thermal conductivity and emissivity. As corrosion progresses, these products migrate into the concrete cover, filling its pores. Corrosion processes are also accompanied by delamination at the concrete-reinforcement interface, as well as the formation of cracks and microcracks in concrete. In bridge structures, these changes are further intensified by cyclic freezing/thawing and the corresponding structural degradation of concrete. All these structural changes disrupt the flow of thermal energy, which is manifested by changes in the concrete surface temperature.

Passive thermography (primarily solar heating) can be used in corrosion diagnostics [133]. However, this method does not generate large temperature gradients, which limits its usefulness in corrosion identification. For this reason, active thermography is most often used, in which the rebar is remotely heated (Figure 14). The contrast between the thermal properties of the steel-concrete composite in corroded and non-corroded areas results in different temperature values, visible in the thermogram. Sequential recording of thermal images allows for the assessment of temperature gradients, the manner and rate of their changes, and thus indirectly identifies corrosion.

Published research results do not indicate a clear trend in temperature change with the development of corrosion phenomena. It is generally believed that corrosion reduces the thermal conductivity of the concrete cover and the immediate surroundings of the bar [121]. However, the complexity of the physical and chemical processes accompanying corrosion can result in different thermal responses of the structural element, depending on the corrosion advancement and the dominant physical or electrochemical phenomenon. Mechanisms that disrupt heat transfer include alkali aggregate reaction, cracks, corrosion products, and other deterioration mechanisms [134].

The usefulness of infrared thermography for detecting corrosion of reinforcing bars in concrete was demonstrated, for example,, in [135]. The tests were conducted using the active method (Figure 14). It was found that general corrosion phenomena (formation and migration of corrosion products, crack propagation) led to an increase in the insulating capacity of the bar-cover system (reduction in the temperature on the concrete surface) in the case of bars without protective coatings. This phenomenon appears to be primarily the result of crack development around the corroded bar, which constitutes a barrier to heat flow.

Previous work [90] also found a decrease in the concrete surface temperature with corrosion progression. At the same time, it was found that increasing corrosion was accompanied by an increase in the temperature of the bar itself (increasing resistance resulting from the reduction in cross-section). This confirms that the formation and migration of corrosion products increased the insulation capacity of the cover. The authors of [90] also indicated that the effectiveness of IRT in detecting corrosion increased with increasing bar diameter and decreasing cover thickness.

**Figure 15:**
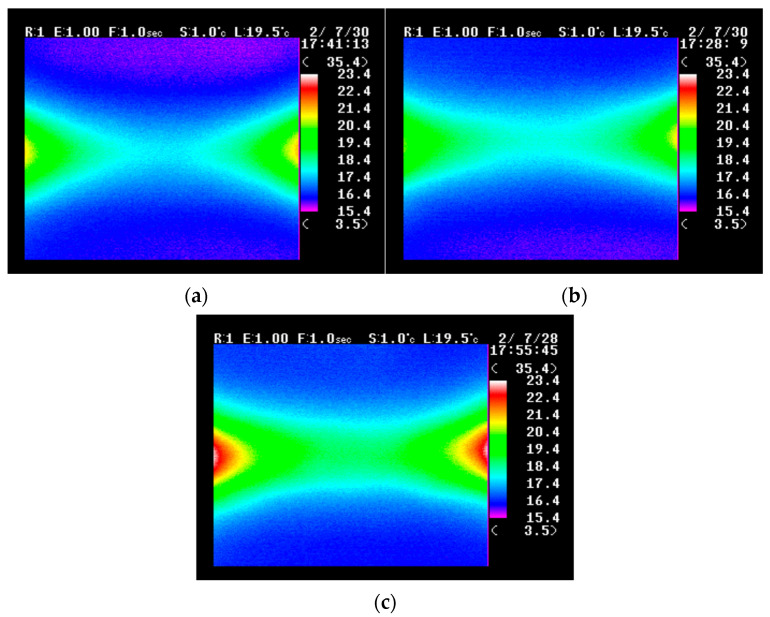
Effect of the reinforcement corrosion on concrete surface temperature after heating the bar for 480 s: (**a**) no corrosion; (**b**) 5% corrosion ratio; (**c**) 10% corrosion ratio, from [136].

The authors of [136] drew opposite conclusions, observing an increase in the temperature on the concrete surface with increasing reinforcement corrosion. In this case, however, the dominant phenomenon was not the thermal insulation of cracks and air voids, but the corrosion itself and the expansion of its products. The observed thermal effect is largely the result of increased heat release associated with the increase in the electrical resistance of the rebar. An example of the relationship between corrosion ratio and concrete surface temperature is illustrated in Figure 15.

Similar observations were described in [36]. As the corrosion degree increased (ranging from 0 to 30%), both the peak temperature on the sample surface and the heating rate increased. A similar, though not as strong, relationship was observed for the cooling rate. The authors attribute these effects to electrochemical property changes, such as electrical resistivity, on the steel rebar surface. A comparative analysis of samples with 1 and 1.5-inch-thick cover also showed that a greater cover thickness reduced this effect, making the use of IRT in these cases difficult and ineffective.

A comparison of the results from [135] and [136] indicates the presence of two competing mechanisms: (i) generation of thermal energy in the reinforcing bar and its increase as a result of the corrosion-induced reduction in the bar cross-section, which implies an increase in its resistance, and (ii) a change in the thermal conductivity of the concrete cover, which is a combined effect of various corrosion changes (formation of corrosion products, their migration in the concrete cover, crack formation, etc.). Depending on the experimental setup and corrosion stage, one of the mechanisms may dominate, resulting in a different response on the concrete surface.

Interestingly, in [135], the phenomenon of reduced insulation and increased surface temperature was observed in epoxy-coated rebars. However, it should be noted that the measured differences were not large, on the order of a few percent, and the applied testing methodology (primarily heating the bar from the inside) largely limited the influence of external factors. It therefore appears that in real-life scenarios, thermography has limited capabilities for corrosion detection.

Despite this, there are studies that successfully utilize IRT in engineering corrosion diagnostics. For example, in [25], practical tests of in-service reinforced concrete columns were conducted. In this case, too, the dominant effect was an increase in the rebar resistance and increased thermal energy generated as a result of corrosion.

The authors of [137] also noted the practical limitations of thermographic detection of corrosion. It was observed that the initial stages of corrosion, prior to crack formation, did not generate sufficient thermal contrast (minimum 0.5 °C) capable of separating corroded and uncorroded areas. Only the formation of diagonal cracks, associated with the increase in the volume of corrosion products, revealed the effects visible in the thermograms. This allowed for the determination of a threshold value distinguishing between reference and corroded areas. The studies described in [137] therefore indicate that the IRT method was effective in detecting only the later stages of corrosion, where the expansion of oxidation products caused the formation of cracks.

Visual evaluation of thermograms also has significant limitations in the detection of corrosion, therefore machine learning methods are constantly developed to support the analysis and interpretation of results [116].

The relationships described above were determined in laboratory conditions, with strictly controlled corrosion development and reinforcement heating. In practical structural inspections, the operator cannot control these factors, and the measurement is further distorted by changing environmental conditions. Furthermore, corrosion itself is accompanied by other factors, such as moisture migration, salt leaching, concrete delamination, and others. The impact of these phenomena on the obtained thermal images is characterized in [32]. By recording temperature changes on the inside of a concrete partition heated by external sun radiation, it was found that the presence of moisture in concrete increases the thermal inertia of the concrete element, which slows down the heating of the concrete by sun radiation, as well as its cooling after sunset. In areas of concrete delamination, higher temperatures were observed during the day than in sound areas, due to local thinning of the element and its lower thermal resistance. The presence of mineral salts resulted in a temperature increase due to the reflectance increase. Although the authors identified a clear impact of these factors on surface temperature readings, when these phenomena occur simultaneously, interpretation of the results is difficult and subject to significant uncertainty. Therefore, it is recommended to combine thermal imaging diagnostics with other research methods, such as ground-penetrating radar (GPR) [32,138].

Concluding from the above, corrosion diagnosis using thermography is complex and presents significant interpretation challenges. Unlike voids and delamination, rebar corrosion generates several competing mechanisms, resulting in opposing IRT responses. These phenomena include the migration of moisture and chlorides into the concrete cover, the oxidation of reinforcing steel, the migration of corrosion products, and the development of cracks and microcracks. When rebar excitation is used, local changes in the electrical resistance, resulting from rebar thinning, introduces additional interference. As a result, both temperature drops and increases in the areas above the corroded rebar are reported in the literature. Published data indicate that thermography does not provide good results in detecting early stages (initiation) of corrosion, but its effectiveness is relatively high in the case of advanced stages of corrosion, when it is accompanied by crack formation and delamination. However, in practical applications, the complexity of the phenomena occurring in concrete makes the use of IRT for corrosion detection difficult and should be supported by other NDT methods.

### 4.4. Diagnostics of External Composite Reinforcements

**Figure 16:**
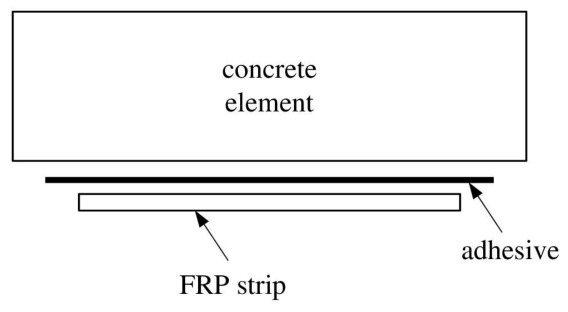
External reinforcement of a concrete element using FRP composite strips.

In recent years, concrete structures have often been reinforced using external reinforcement made of FRP composite mats or strips. This type of reinforcement involves gluing the composite element to the concrete surface (Figure 16), with optional mechanical anchoring.

The IRT method is an effective tool for detecting subsurface defects in such reinforcements. This applies to defects in the composite itself [6,58,139], the composite-concrete bond [140], as well as subsurface defects in the concrete beneath the reinforcement surface [45,46,108].

Thermographic defect detection in FRP reinforcements in concrete is a relatively complex issue, as the reinforced concrete element with FRP reinforcement system consists of several materials with different thermal properties: concrete, adhesive, fibers, and resin. Furthermore, the composite itself exhibits different properties depending on the heat flow direction considered (along and across the fibers). For example, the thermal diffusivity of CFRP fibers is high, on the order of 240–280.2 × 10^−6^ m^2^/s. This results in a very rapid drop in surface temperature after the heat source is removed. However, diffusivity in the perpendicular direction is approximately 100 times lower, resulting in a significantly slower material response.

At the same time, the resin is characterized by high insulating properties (for example, the emissivity of epoxy resin is about 0.7–1.1 × 10^−7^ m^2^/s [111]).

Despite the complexity presented here, the key point is that the thermal conductivity of resin and concrete is still significantly higher than that of air-filled voids/delaminations, making defective areas clearly visible in the thermogram. In the case of active thermography, most often used in this case, these areas exhibit elevated temperatures, creating so-called hot spots.

**Figure 17:**
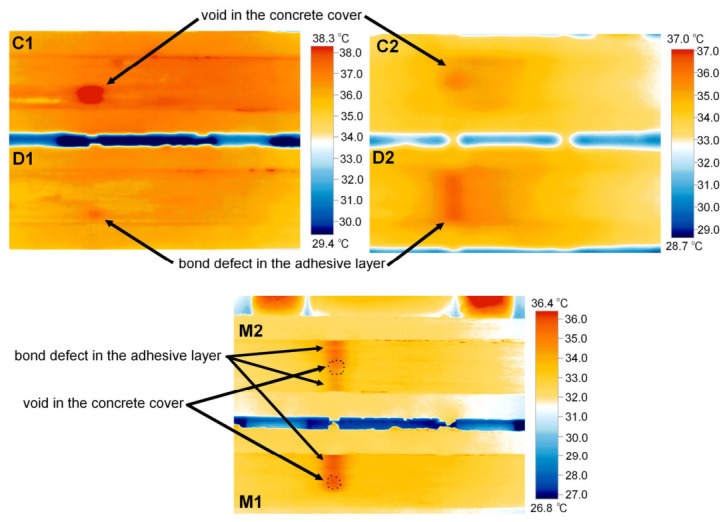
Effectiveness of active IRT for defects detection under CFRP strengthening strips: C1, C2—thermogram of the strip over a void in concrete; D1, D2—temperature distribution on the strip surface at the defect in the adhesive layer; M1, M2—thermographic image of the strip at simultaneous occurrence of adhesive defect and voids in concrete, from [28].

The usefulness of active IRT for identifying structural defects in CFRP (Carbon Fiber Reinforced Polymer) strip reinforcement was analyzed in [28]. The method demonstrated high efficiency and accuracy in detecting various defects in the concrete and adhesive layer (Figure 17). Samples heated under laboratory conditions were analyzed. Controlled defects were introduced into the samples, including holes in the concrete (thermograms C1 and C2 in Figure 17), adhesive layer defects (thermograms D1 and D2), and both types of defects simultaneously (M1 and M2). As can be seen in Figure 17, in virtually every case, the defects were clearly visible in the thermogram as areas of elevated temperature.

However, it was observed that after removing the heat source, the temperature gradient quickly disappeared, therefore this method allows for a rough assessment of the defect size. The ability to distinguish between individual defect types and detect complex defects and those located at greater depths was also limited.

In [108], integrated infrared thermography and deep learning techniques were used to detect defects in CFRP reinforcements. The additional inclusion of RGB imaging and training the model using an extended dataset allowed for achieving an efficiency of the entire procedure of 96%. As noted by the authors, the integration of thermography with deep learning provides a tool for fast, efficient, effective, and precise inspection of CFRP reinforcements.

As the above examples demonstrate, thermography presents high sensitivity in detecting defects in FRP reinforcements, particularly such as delaminations, discontinuities in the adhesive layer, or voids in the concrete beneath the reinforcement surface. Due to the high contrast in thermal conductivity between voids and the concrete/composite, active thermography provides particularly good results. However, a practical limitation in interpreting results is the anisotropy of the thermal properties of FRP composites and, above all, the rapid temperature equalization after heating, which results in the risk of undetected defects and very limited possibilities for estimating their dimensions.

### 4.5. Assessment of Material Properties

Thermographic methods can be used both to assess the material properties of hardened concrete and to monitor its setting process.

Hong et al. [22] used passive IRT technology to assess the quality and detect defects in early-age concrete. Under semi-adiabatic conditions, IRT enabled seamless detection of near-surface voids.

IRT tracking of concrete temperature changes during curing can be used to predict its strength development. In [135], using deep-learning-based image segmentation and maturity-based equation, concrete strength changes were determined with an accuracy of up to 80%.

The IRT methodology can be used to assess damage and operational defects in concrete, for example, after exposure to fire temperatures. This issue was analyzed, for example, in [77]. As noted, thermal imaging is an effective tool for identifying damaged areas, regardless of the concrete’s moisture content prior to the fire. However, the IRT response depends on the temperature range reached by concrete, which in turn implies specific destructive mechanisms. Study [77] indicated that at temperatures below 400 °C, moisture contained in concrete can absorb some thermal energy. Thermograms taken after the sample was cooled down show a reduction in the mean temperature change. Temperatures of 600 °C and higher resulted in chemical decomposition of concrete, which became a key factor in determining the mean temperature changes in the thermogram.

The IRT method is used to detect leaks and other areas of increased moisture. In the context of concrete structure diagnostics, this most often indicates water migration through cracks, for example, in retaining structures and foundations. Due to water evaporation, lower temperatures are observed in these areas on thermograms [49]. To improve the readability of results and facilitate their interpretation, data processing is performed using various statistical and numerical methods, such as descriptive statistics, thermal image subtraction, or determining the thermal index, related to the cooling rate [141].

Generally, among the material parameters analyzed in the literature, variable concrete moisture content is a feature particularly well visible in thermographic images. The presence of moisture alters thermal inertia and of evaporation processes, which manifests itself in areas of visibly lower temperature. However, it should be remembered that the presence of moisture can mask defects in thermographic images due to the partial uniformity of heat transfer conditions.

### 4.6. Comparative Synthesis of Thermographic Defect Detectability

The examples mentioned above clearly demonstrate that thermography is an effective NDT method for diagnosing concrete structures. However, its effectiveness varies depending on the type of defect being investigated. This is primarily related to its material parameters, where a high contrast in thermal parameters between the defect and the bulk material is desirable. The dimensions and shape of the object, as well as the environmental conditions, are also crucial. Table 6 summarizes the basic conclusions from the studies discussed in Section 4.1, Section 4.2, Section 4.3, Section 4.4 and Section 4.5.

Comparative analysis indicates that the best detectability is achieved for all air defects, especially shallow ones such as delaminations, voids, and debonding of FRP reinforcements. This is due to the significant disruption of heat transfer through air, which is characterized by low thermal conductivity. Good detectability in these cases is achieved not only with active thermography but also, under favorable conditions, with passive thermography.

Thermography offers significantly fewer possibilities for detecting narrow cracks, deeply embedded reinforcement, or early stages of corrosion. In these cases, the thermal contrast is low. In the case of corrosion, interpretation difficulties arise due to the complexity of the phenomena occurring in corroding concrete.

The presented results also suggest that active thermography is generally more effective than passive approaches, especially for deeper defects. However, in practice, thermal excitation is only possible on small areas, so diagnostics of large surfaces (such as bridge slabs) are typically performed using passive methods.

It should also pointed out that thermography does not detect defects themselves, but the accompanying physical mechanisms. Table 7 summarizes the basic types of objects and phenomena detected using IRT, along with the corresponding physical mechanisms that generate thermal effects. This means that similar effects in a thermogram may be the result of different phenomena, which complicates the interpretation.

Air voids are particularly visible in thermograms, but debonding or crack formation can also produce a similar effect. Reinforcement corrosion can also generate voids and delaminations, so similar temperature distributions may be the result of different destructive mechanisms.

The resulting ambiguities in interpretation mean that the interpretation of IRT results should be conducted with great caution and supported by other diagnostic methods.

### 4.7. Some Aspects of Data Processing

As mentioned before, thermography is a research technique with great potential and a wide range of applications in the diagnosis of concrete structures. Despite this, there is currently no single, consistent research procedure. This primarily applies to data analysis methods, which allow for the reduction or elimination of random factors that interfere with measurements, and secondly, through the use of artificial intelligence (AI), enable the extraction of valuable information from complex data sets. The use of AI opens up new possibilities for automatic pattern recognition, defect categorization, and improved interpretation of low-contrast data. While these techniques do not eliminate the physical limitations of the method, they do improve defect detection and overall method reliability.

Therefore, in recent years, the main research directions have included the development of rapid and effective procedures for interpreting thermograms for defect detection.

Regardless of the methods and algorithms used, processing includes 4 basic stages [82]:

- Data acquisition;
- Preprocessing and enhancement;
- Segmentation and feature extraction;
- Classification and quantification.

Traditionally, data analysis involves visual interpretation of temperature distribution patterns. However, this method is subjective, unreliable, and requires extensive experience.

Interpretation based on thermal contrast is more objective, as it is based on a defined temperature difference (ΔT) between the sound and the damaged concrete. However, in this case, the contrast may be influenced by other random factors (concrete surface structure, environmental factors, etc.), which means that in practical situations the interpretation is subject to significant error [72,142]. To reduce these limitations, various enhancement methods are used. They improve the signal-to-noise ratio while preserving key diagnostic information. It should be noted that these methods do not detect defects independently but primarily serve to improve data quality and resolution. Various approaches have been discussed in the literature, such as contrast enhancement (e.g., by histogram stretching, equalization [143]), local methods—CLAHE [144]), noise filtration (e.g., using Gaussian filters [79], median filters [75], and others), space-frequency transformations (e.g., FFT—Fast Fourier Transform [78]), and dynamic methods (for sequential measurements). Despite the significant practical importance of these methods, their inappropriate application can lead to negative consequences, such as the risk of artificial noise amplification; their effectiveness and usefulness often depend on correct parameter calibration.

Another effective image processing method is segmentation, which involves automatic division of the thermogram into areas of similar temperature, which enables the identification of areas that differ from the background and are therefore “suspected” of having a defect [145].

The influence of random factors can be limited to some extent by using image processing techniques [144], which most often use threshold values and gradients. In general, these methods involve converting an IRT image into a binary image (sound and damaged areas) based on an assumed threshold/cutoff value. Basic variants include global thresholding (automatic threshold determination by maximizing interclass variance) and adaptive thresholding, effective for non-uniform heating [82]. The advantages of this procedure include computational simplicity, speed, and ease of interpretation. The disadvantage of threshold-based procedures is the practical difficulty in defining the threshold value, especially under different weather conditions [146,147].

This problem was analyzed in [7]. Using passive thermography, temperature distributions on the surface of a reinforced concrete bridge slab were determined. Temperature distributions in the delaminating slabs were determined using finite element simulation. Comparative analysis of both distributions enabled the determination of the limiting value and the detection of delamination. The authors also noted the importance of data processing for the quality of results and accurate prediction of defects.

In the analysis of delamination of concrete elements, morphological operations such as opening, closing, and erosion are also efficient [82].

Another technique effective in identifying delamination is thermal image clustering. It involves automatically combining pixels with the same temperatures into clusters, without prior user input of reference data. Among the clusters thus determined, the algorithm identifies areas with temperatures different from the reference temperature, which may indicate the presence of defects [145]. The advantage of this method over the previously mentioned techniques is its objective nature, meaning that the results are not dependent on the operator’s experience.

Combined methods are also described in the literature. For example, Ichi and Dorafshan [144] proposed an image segmentation procedure with clustering elements, in which the model automatically optimizes parameters (e.g., temperature threshold) and uses performance metrics to select the best solution.

Feature extraction techniques involve transforming a raw thermogram (or sequence of thermograms) into a set of numerical features that allow for the identification and quantification of characteristic thermal signatures that describe important image properties, such as temperature distribution, texture, or anomaly shape. This approach is more comprehensive and increases robustness to noise, but it introduces significant computational and interpretation complexity. Its application requires significant experience and expert knowledge. Basic variants of this technique include statistical feature analysis [42,148], as well as systematic decomposition, and frequency domain analysis [149,150].

In recent years, the development of machine learning (ML) methods has opened up new perspectives in the analysis of thermographic data and interpretation of results. ML methods, depending on their approach, are divided into several categories.

In the supervised learning approach, a model is trained on data containing both input information and the corresponding correct outputs (labels). The goal is to teach the model the relationship (mapping) between input and output data so that it can correctly predict the correct outputs for new, unknown inputs in the future. The training process itself involves minimizing the error between the model’s predictions and the actual outputs. Typical tasks in supervised learning include classification and regression.

One of the most commonly used, yet simple and effective, supervised algorithms is the k-nearest neighbor (KNN) method. In this algorithm, for each new observation, the k most similar examples in the training set are found and then the output is calculated based on these results. KNN does not require the construction of an explicit model, and its effectiveness depends on the selection of parameter k, the features incorporated, and the quality of the training data.

The KNN algorithm was used, for example, in the paper mentioned above [144] to identify delamination areas of a bridge deck.

Another variant of supervised learning algorithms is tree-based methods, which gradually divide data into increasingly uniform subsets (branches), effectively creating a tree-like structure. These methods offer the advantage of ease of interpretation and the absence of data scaling, but individual trees are susceptible to overfitting. These methods are divided into subtypes, the most common of which are decision tree (single tree), random forest, extremely randomized tree, extreme gradient boosting, adaboost (adaptive boosting), and gradient boosting (sequential model improvement).

For example, Park et al. [87] combined passive thermography and selected supervised learning algorithms to determine crack depth in concrete. Of the four algorithms used (decision tree, extremely randomized tree, gradient boosting, and adaboost), the latter proved to be the most effective in assessing crack geometry. As the authors noted, the methodology used was effective in quickly assessing the degree of cracking in large-area elements.

Within the supervised learning group, neural network-based models are also employed. These models consist of layers of interconnected neurons that process data through weighted sums and nonlinear activation functions. Learning involves iteratively updating weights to minimize differences between the model’s predictions and the training (real) data. Neural networks are well-suited to classification problems in complex datasets, such as thermographic images. In the field of thermographic diagnostics of concrete structures, neural networks are primarily used to identify defective areas and locate reinforcement [55].

One of the simplest forms of supervised learning is linear regression-based models, which assume a linear relationship between the input data and the output. As before, model training involves adjusting the coefficients to minimize the differences between the predicted and actual values (usually using the least squares method). These models are computationally efficient and easy to interpret, but their effectiveness depends on meeting the assumption of a linear relationship between the data.

Unsupervised techniques involve determining the internal structure of input data without an additional reference base, in the absence of unknown output. The goal of such procedures is to discover the structure of the analyzed data, relationships, or patterns, through comparative analysis of various parameters (features) and grouping similar observations. These methods are particularly useful in the absence of reference data. The k means algorithm is most commonly used in assessing the effects of IRT-based design, for example, to assess the degree of delamination [121]. The principle of this algorithm involves grouping data into k separate clusters, within which the data are as similar as possible while maintaining the greatest possible differences between individual clusters. Advantages of this method include good performance on large data sets and ease of implementation. Disadvantages include sensitivity to outliers and the need to predefine the number of clusters (k).

Deep learning models and convolutional neural networks (CNNs) are also widely used in thermographic analyses. The advantage of these solutions is no need to define the analyzed features, as the model independently extracts key information from the dataset. Thanks to the large number of layers and parameters, these models can represent very complex, nonlinear relationships, but they require large data sets and significant computing power to operate effectively. This defined model structure makes them highly effective in solving problems such as classification, segmentation, and detection.

Transfer learning (TL) is an approach in which a model uses knowledge acquired from one task to solve another one. Such a model is pre-trained on one task and then adapted to a new one. In practice, this involves using previously trained layers as feature extractors and, if necessary, fine-tuning them on new data. TL works most effectively when the tasks are similar and the data has a similar structure, making it a well-suited solution for thermographic data analysis.

The efficiency and usefulness of TL in thermographic diagnostics of concrete has been demonstrated, for example in [151]. The model was characterized by high efficiency of detecting crack, spalling, and potential subsurface defects.

The TL approach to CNN training was also described in [152], where it was found that the highest efficiency was achieved by analyses based on combined thermographic and visible images.

In [153], a data acquisition and post-processing procedure was proposed that allows for reducing the impact of environmental disturbances. It is based on the concept of Principal Component Thermography (PCT), a processing procedure based on Principal Component Analysis (PCA). It involves transforming a sequence of thermograms (as a function of time) into a new space in which the dominant temperature change patterns are extracted. This allows for reducing environmental disturbances and highlighting/emphasizing data generated by material defects.

A practical limitation in the use of machine learning models is the difficulty of obtaining reliable training data. This requires complex and challenging experimental research, hence the development of procedures for generating synthetic data, which can be used to train networks. One option is the use of data augmentation procedures, which essentially involve increasing the amount of training data by generating new examples based on existing ones, without the need to generate new measurement data. In practice, this involves applying various data transformations while preserving their physical meaning. Most often, these include simple geometric transformations (such as scaling), brightness and contrast changes, adding noise, or transformations related to temperature distribution.

A proposal to solve the problem of data scarcity using data augmentation was presented, for example, by Ali and Cha in [154], where training data were generated synthetically using an attention-based generative adversarial network (AGAN). The model trained in this way was characterized by an efficiency exceeding 90%.

Another current research problem is reducing the inherent limitations of the method (primarily sensitivity to measurement conditions and the influence of the emissivity of the tested surface) by fusion of IRT data with results obtained using other research methods (multimodal fusion). In the literature, IRT results are most often combined with GPR, ultrasonic techniques, and electrochemical methods. This research procedure combines information about the temperature field with data on the structure and properties of the material, making data interpretation more reliable while improving diagnostic sensitivity and selectivity.

Another approach is image data fusion, which most often involves combining thermograms with RGB images (visible data) or fusing thermographic images taken at different time intervals. This enables the assessment of the dynamics of changes in temperature distributions, which translates into better differentiation of defects and artifacts (like surface contamination).

Data fusion itself can occur at various levels. One option is feature-level fusion, where features are extracted separately from the results of each research method and then combined into a single vector, which forms the basis for creating a decision rule. The advantage of such an algorithm is the ability to capture complex relationships between features from different methods, thus ensuring high diagnostic efficiency.

In decision-level fusion, all sources (research methods) are analyzed independently [21], leading to separate decisions within each method. All these decisions are then aggregated, for example, by majority voting or by using the average of probabilities. The advantage of this method is flexibility and robustness to errors of a single data source, but at the cost of not being able to assess correlations between source data, which may result in lower accuracy.

## 5. Conclusions

Infrared thermography (IRT) serves as a robust, full-field diagnostic tool for the structural health monitoring of reinforced concrete, provided that interpretation is grounded in heat transfer physics rather than surface temperature observations alone. This review establishes that defect detectability is fundamentally governed by the interplay of thermal diffusivity, lateral heat flow, and environmental boundary conditions.

The quantitative synthesis of the state-of-the-art leads to the following key conclusions:

- Dependable defect identification requires a minimum thermal contrast of 0.5 °C to overcome the signal-to-noise limitations inherent in heterogeneous concrete matrices. While passive IRT typically generates contrasts within the 0.5–2.0 °C range under solar loading, active thermography is necessary for quantifying features at depths of 5–10 cm.
- Concrete moisture content is a critical source of measurement error; a variation in moisture as low as 1.5% can induce emissivity shifts exceeding 10%, which may mask subsurface anomalies or produce false-positive indications.
- Air-filled discontinuities, such as delaminations and FRP debonding, exhibit the highest detectability due to the significant thermal resistance of the air gap. Conversely, the detection of narrow cracks and early-stage corrosion remains challenging due to rapid thermal equalization and low contrast, requiring active excitation and advanced signal processing.
- This work resolves the ambiguity between different diagnostic targets by distinguishing their physical signatures: rebar location is identified through periodic patterns of thermal inertia, whereas corrosion is characterized by the competition between resistive heating (increased temperature) and the insulating effect of corrosion-induced cracks (decreased temperature).
- Artificial Intelligence, specifically CNNs and Transfer Learning, significantly enhances the interpretation of low-contrast data and automates defect categorization. However, the reliability of these models depends on their integration into a physics-oriented framework that accounts for the probabilistic nature of thermal wave attenuation with depth.

In conclusion, moving beyond qualitative imaging toward quantitative, physics-based diagnostics is essential for the next generation of autonomous SHM systems. Future research should prioritize the standardization of detectability criteria and the development of multimodal data fusion strategies (e.g., IRT-GPR) to overcome current penetration depth and resolution limits.

## Figures and Tables

**Table 1:** Comparison of the novelty of the present work with existing literature reviews.

Comparative Criterion	Conventional Literature Reviews	This Work (Present Review)
Main Approach	Primarily focus on descriptive case studies or general applications of NDT methods.	Physics-oriented framework—directly linking diagnostics to fundamental heat transfer mechanisms (radiation, convection, and conduction).
Detectability Criteria	Often subjective, relying on qualitative visual interpretation of thermograms.	Definition of quantitative detection thresholds, such as the minimum thermal contrast requirement of 0.5 °C.
Environmental Analysis	General mentions of solar loading or wind effects without quantification.	Detailed analysis of moisture impact on concrete emissivity (variations exceeding 10%), which critically affects measurement accuracy.
Role of AI	Treating AI as an autonomous or “black-box” image processing tool.	Holistic integration—linking AI algorithms (CNN, KNN, Transfer Learning) with physical limits like thermal diffusivity and thermal wave length.
Method Limitations	Providing approximate or purely empirical ranges of application.	Precise definition of spatial and thermal resolution limits and penetration depths (typically 5–10 cm) based on excitation parameters.
Material Scope	Usually limited to a single material type or general civil infrastructure.	Multi-material analysis of reinforced concrete and composite reinforcements (FRP/CFRP), including the influence of carbon nanotubes (CNTs) on thermal contrast

**Table 2:** Typical thermophysical properties of selected engineering materials relevant to infrared thermography.

Materials	Thermal Conductivity (W/mK)	Thermal Diffusivity (m^2^/s)	Heat Capacity (J/kgK)	Typical Emissivity	Implications for Thermographic Inspection	Source
aluminum	~200–237	~8–10 × 10^−5^	~900	0.05–0.10	Extremely high thermal diffusivity reduces thermal contrast; surface preparation (e.g., coatings) is often required to improve emissivity and detection sensitivity.	[14]
CFRP composite	5–10 (anisotropic)	~1 × 10^−6^	750–900	0.80–0.90	Anisotropic conduction influences defect visibility; delaminations often produce strong thermal contrast.	[14,91,92]
GFRP composite	0.25–0.6 (anisotropic)	(1–3)~10^−7^	800–1000	0.85–0.95	Low thermal conductivity enhances thermal contrast, while anisotropic heat conduction influences defect visibility depending on fiber orientation.	[14,91,93]
concrete	~1.4–2.0	~3–5 × 10^−7^	~880	0.90–0.95	Low thermal diffusivity allows relatively deep defect detection; heat propagates slowly, enabling clear thermal contrast during pulsed or lock-in thermography.	[94]
reinforced concrete	1.7–2.5	~4 × 10^−7^	~900	0.90–0.95	Steel reinforcement alters heat flow and may produce characteristic thermal patterns during inspection.	[4]
steel	~45–60	~1.0–1.7 × 10^−5^	~470	0.20–0.80	High thermal diffusivity leads to rapid heat dissipation, limiting defect detection depth and requiring high-speed thermal excitation or lock-in methods.	[14]
polymer (epoxy)	0.20–0.30	~1 × 10^−7^	1200–2000	0.90–0.95	Slow heat diffusion increases thermal contrast but may require longer observation times.	[14]
copper	380–400	~1.1 × 10^−4^	~385	0.03–0.05	Very high conductivity causes rapid heat spreading, making subsurface defects difficult to resolve.	[91]
ceramic (Al_2_O_3_)	20–30	~1 × 10^−5^	~880	0.70–0.90	Moderate conductivity allows crack detection, though thermal gradients decay relatively quickly.	[4]
asphalt	0.7–1.0	~2 × 10^−7^	~920	0.90–0.98	High emissivity and low diffusivity favor passive thermography in pavement inspections.	[4]

**Table 3:** Correlation between the microstructural features observed in the SEM analysis and their direct impact on the thermophysical parameters governing infrared imaging.

Microstructural Feature (from Figure 1)	Physical Observation	Impact on Thermal Parameters	Effect on IR Thermography
Strong Interfacial Adhesion (Figure 1b)	Excellent bonding between CNTs and epoxy matrix.	Lower thermal contact resistance. Increases effective (k).	Faster heat dissipation and higher thermal stability.
CNT Alignment (Figure 1e)	Nanotubes oriented in a specific direction.	Anisotropic thermal conductivity. Creates “heat highways”.	Directional heat flow, allowing for better depth resolution.
Interfacial Debonding (Figure 1c,d)	Separation of fibers/matrix at high temperatures (100 °C).	Acts as a thermal barrier. Local decrease in effusivity (e).	Appears as “hot spots” or anomalies in the thermal contrast.
Microcrack Formation (Figure 1f)	Structural damage occurring under thermal stress.	Significant disruption of heat flow. Increases scattering of phonons.	Enhanced detectability of early-stage degradation.

**Table 4:** Typical emissivity values of concrete surfaces in the long-wave infrared (LWIR) range under different environmental and surface conditions.

Surface Condition	Typical Emissivity ε (−)	Physical Explanation	Implication for Thermography	Refs.
Dry, mature concrete	0.90–0.94	Stable microstructure, reduced surface moisture	Baseline condition; relatively stable measurements	[67]
Wet/saturated concrete	0.96–0.98	High emissivity of water; pore filling	Higher apparent temperature; reduced sensitivity to reflection	[64]
Drying surface (transient state)	0.92–0.96 (variable)	Changing pore structure and moisture distribution	Time-dependent measurement error; apparent temperature drift	[20,64]
Rough/aged surface	0.93–0.97	Increased diffuse radiation due to roughness	Improved radiative uniformity; slightly higher ε	[20,68]
Surface with efflorescence/contamination	0.88–0.95	Altered surface composition and radiative properties	Increased uncertainty; potential local contrast artifacts	[66,100]

**Table 5:** Comparison between active and passive thermographic methods.

Criterion	Passive Thermography	Active Thermography
Applicable Conditions	Large-scale in situ inspections (bridges, building façades, dams) where external heating is impractical [71].	Laboratory settings or controlled field inspections of specific structural components [55,59].
Excitation Requirements	Naturally occurring thermal gradients (e.g., solar radiation, diurnal cycles, operational loads) [61].	Controlled external sources (e.g., flash lamps, halogen heaters, induction coils, or microwaves) [61].
Detectable Defect Types	Large-scale delaminations, air/water-filled voids, and significant moisture ingress [31,117].	Near-surface cracks, voids, honeycombing, CFRP debonding, rebar distribution, and early-stage corrosion [18,54,60].
Typical Depth Range	Generally limited to near-surface features (approx. 2–5 cm) due to weak natural contrasts [48].	Adjustable via excitation parameters; typically effective up to 5–10 cm in concrete [28,52,54].
Main Advantages	Low cost, simplicity, no disruption to traffic or structure use, and high scalability for large assets [26,50,118].	High thermal contrast, improved signal-to-noise ratio, repeatable results, and controllable penetration depth [53,118].
Main Limitations	Weak thermal contrast (0.5–2 °C), low repeatability, and heavy dependence on daily weather cycles [26,84,101].	Higher cost and complexity, risk of surface overheating, and high energy demand for large concrete sections [11,26,54].
Sources of False Detection	Solar shading, clouds, wind-induced cooling, surface reflections, and oil stains (emissivity artifacts) [51].	Non-uniform heating, complex defect geometries (S/D ratio < 3), and localized moisture variations [11,18].

**Table 6:** The usefulness of thermography in various aspects of reinforced concrete diagnostics.

Damage Type	Detectability	Preferred Mode	Main Limitation
delamination	high	passive/active	depth/moisture
voids	high	passive/active	size-to-depth ratio
cracks	moderate	active	narrow width
rebar	moderate	active	cover thickness
corrosion	low/moderate	active	complex thermal response
moisture	high	passive	interference with other defects
FRP debonding	high	active	rapid thermal equalization

**Table 7:** Comparison of thermal phenomena related to the diagnostic target and potential interpretation overlap.

Diagnostic Target	Dominant Thermal Mechanism	Potential Overlap
delamination	air-gap insulation	debonding
rebar	heat flow disturbance	moisture, shallow voids
corrosion	cracking, resistive heating	delamination, moisture
FRP debonding	interfacial thermal barrier	delamination
moisture	evaporation, thermal inertia	corrosion

## Data Availability

No new data were created or analyzed in this study. Data sharing is not applicable to this article.
